# Comparative transcription analysis of photosensitive and non-photosensitive eggplants to identify genes involved in dark regulated anthocyanin synthesis

**DOI:** 10.1186/s12864-019-6023-4

**Published:** 2019-08-28

**Authors:** Yongjun He, Hang Chen, Lu Zhou, Yang Liu, Huoying Chen

**Affiliations:** School of Agriculture and Biology, Shanghai JiaoTong University, 800 Dongchuan Road, Minhang District, Shanghai, 200240 China

**Keywords:** Eggplant (*Solanum melongena* L.), Non-photosensitive, Dark-regulated, Transcriptome, Anthocyanin biosynthesis

## Abstract

**Background:**

Light is a key environmental factor in regulation of anthocyanin biosynthesis. Through a large number of bagging screenings, we obtained non-photosensitive eggplants that still have decent amount of anthocyanin synthesized after bagging. In the present study, transcriptome was made to explore the molecular mechanism of dark-regulated anthocyanin synthesis in non-photosensitive eggplant.

**Results:**

The transcriptome of the pericarp at 0 h, 0.5 h, 4 h, and 8 h after bag removal were sequenced and analyzed. Comparison of the sequencing data with those of photosensitive eggplant for the same time period showed that anthocyanin synthesis genes had different expression trends. Based on the expression trends of the structural genes, it was discovered that 22 transcription factors and 4 light signal transduction elements may be involved in the anthocyanin synthesis in two types of eggplants. Through transcription factor target gene prediction and yeast one-hybrid assay, SmBIM1, SmAP2, SmHD, SmMYB94, SmMYB19, SmTT8, SmYABBY, SmTTG2, and SmMYC2 were identified to be directly or indirectly bound to the promoter of the structural gene *SmCHS*. These results indicate that the identified 9 genes participated in the anthocyanin synthesis in eggplant peel and formed a network of interactions among themselves.

**Conclusions:**

Based on the comparative transcription, the identified 22 transcription factors and 4 light signal transduction elements may act as the key factors in dark regulated anthocyanin synthesis in non-photosensitive eggplant. The results provided a step stone for further analysis of the molecular mechanism of dark-regulated anthocyanin synthesis in non-photosensitive eggplant.

**Electronic supplementary material:**

The online version of this article (10.1186/s12864-019-6023-4) contains supplementary material, which is available to authorized users.

## Background

Anthocyanins are a class of secondary metabolites that contribute to the red, blue, and purple colors in a range of flowers and fruits [1]. Anthocyanins play an important role not only in plant physiology, pollinators and visual appeal of seed communicators, but also in human health, such as preventing cardiovascular disease, controlling obesity and reducing diabetes [2, 3]. Anthocyanin biosynthesis in plants is affected by many factors, including light, temperature, sugar and hormones, with light being the most important [4–7].

Light signaling plays a pivotal role in the control of plant metabolism, growth and development. Light signals are perceived by photoreceptors including phytochrome (PHYA-PHYE), cryptochromes (CRYs), phototropins (PHOTs) and UV resistance locus 8 (UVR8, [8–12]). CRYs are blue light receptor proteins of which some functions have been reported, such as the blue light-dependent hypocotyl inhibition, floral initiation in Arabidopsis, and anthocyanin accumulation in eggplant [13–15]. Studies showed that BIC (blue-light inhibitor of cryptochromes) binds to CRY2 to suppress the blue light–dependent dimerization, photobody formation, phosphorylation, and degradation, resulting in the reduction of physiological activities of CRY2 and thus the inhibition of photomorphogenesis [16]. Phototropins are blue light receptors that control many responses to optimize photosynthesis efficiency. It was shown that *FaPHOT2* played a role in sensing blue light and mediating anthocyanin biosynthesis in strawberry fruits [17]. The phytochrome family of photoreceptors (phyA–phyE) monitor the environment for informational light signals and induce plant growth and developmental responses appropriate to the prevailing conditions. Arabidopsis phytochrome regulates the accumulation of anthocyanins under nitrogen, phosphorus, and cold induction conditions [18, 19]. UVR8 was identified originally as a UV-resistance gene shown to contribute to the UV-B-induced flavonoid accumulation and UV-B protection. In Arabidopsis *uvr8* mutant, *CHS* expression and flavonoid content were reduced significantly [19, 20].

After the photoreceptors perceive light signal, they will further regulate the expression of transcription factors (TFs), such as MYBs, bHLHs, WD40, thereby activating or inhibiting the expression of structural genes and ultimately affecting anthocyanin biosynthesis. COP1 is an E3 ubiquitin ligase that promotes the ubiquitination and degradation of many genes that are related to light signal transmission [13, 21–25]. In addition, COP1 can interact with suppressor of PHYA (SPA) to inhibit photomorphogenesis, while *spa* mutants exhibited characteristics similar to those of *cop1* mutants under dark conditions in Arabidopsis [26–28]. The TFs that control anthocyanin biosynthesis include those of the v-myb avian myeloblastosis viral oncogene homolog (MYB), the basic helix-loop-helix (bHLH), and the Trp-Asp forty amino acid repeat (WD40) proteins [29, 30]. MYB TFs, especially the R2R3-MYB class, play a key role in anthocyanin biosynthesis. The functions of R2R3 MYB TFs in anthocyanin accumulation had been well studied, such as *AtMYB75* in Arabidopsis, *MdMYB1* in apple, *NnMYB5* in *N. nucifera*, *PyMYB10* in *Pyrus pyrifolia*, etc. [31–34]. The bHLHs family can be divided into 26 subgroups, of which subgroup IIIf is associated with flavonoids [35]. In Arabidopsis, *AtTT8*, *AtGL3* and *AtEGL3* have been characterized to positively regulate anthocyanin synthesis [36–39]. In addition, the bHLHs were found to regulate anthocyanin synthesis in other plant species, such as *MdbHLH3* in apple, *LcbHLH* in *Litchi chinensis*, *MtbHLH* (*MtTT8*) in *Medicago truncatula*, and *VvbHLH* (*VvMYC1*) in grape [29, 40–43]. Other transcription factors, including Elongated Hypocotyl 5 (HY5), TCP, and squamosa promoter-binding protein-like 9 (SPL9), have also been shown to be associated with anthocyanin biosynthesis [44–47].

Anthocyanin biosynthesis is regulated by transcription factors that induce the expression of structural genes coded for enzymes in the biosynthesis pathway [48, 49]. Anthocyanins are synthesized from phenylalanine. The synthesis was catalyzed first by phenylalanine ammonia lyase (PAL) and 4-coumarate-CoA ligase (4CL), and then by early flavonoid biosynthetic genes (EBGs) and late biosynthetic genes (LBGs) [50]. The EBGs encode enzymes such as chalcone synthase (CHS), chalcone isomerase (CHI), and flavanone 3-hydroxylase (F3H). The LBGs include dihydroflavonol reductase (DFR), anthocyanidin synthase (ANS), flavonoid 5-Oglucosyltranferase (5GT) and flavonoid 3-Oglucosyltranferase (3GT), which regulate the production of anthocyanins [50].

Eggplant (*Solanum melongena* L.) is a horticultural crop of high economic value and has more than one thousand years of plantation history in China. Greenhouse cultivation and low light conditions, however, often lead to poor coloration of eggplant and reduce its quality. In this study, we obtained non-photosensitive eggplant through a large number of bagging screenings that still synthesized a significant amount of anthocyanin after bagging. Twelve RNA samples of non-photosensitive eggplant before and after light induction were sequenced with the Illumina Hiseq platform. Through comparative analysis of transcriptome data of both photosensitive and non-photosensitive eggplant samples at the same time points, we aimed to find key genes that took part in dark-regulated anthocyanin biosynthesis in non-photosensitive eggplant.

## Results

### Dark treatment and determination of anthocyanin content

**Fig. 1:**
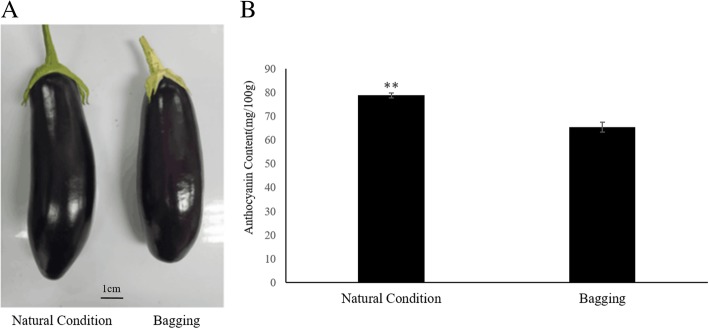
Phenotype and Anthocyanin Content of Non-photosensitive Eggplant under Natural Condition and Bagging. (**a**) Image of non-photosensitive eggplants (**b**) The anthocyanin content. The error bar indicates the standard deviation, based on three biological replicates. Asterisks indicate significant differences, according to Student’s t-test (**, *P* < 0.01)

Our previous study showed that the peel of photosensitive eggplant was white when it was bagged, while the anthocyanin content of peel gradually increased after bag removal [15]. Non-photosensitive eggplant, on the other hand, can synthesize anthocyanins under dark conditions. After the non-photosensitive materials were bagged for 24 days, the color of their pericarp was almost the same as that of the un-bagged eggplant (Fig. 1a). The anthocyanin contents of the un-bagged and the bagged eggplants were 78.7 mg/100 g and 65.4 mg/100 g, respectively. Variance analysis of the anthocyanin content showed that darkness also affected the anthocyanin biosynthesis in non-photosensitive eggplant; however, the eggplant was evenly colored and the fruit attributes were not affected (*P* value = 0.0012) (Fig. 1b).

### De novo assembly and analyses of RNA-Seq data

**Fig. 2:**
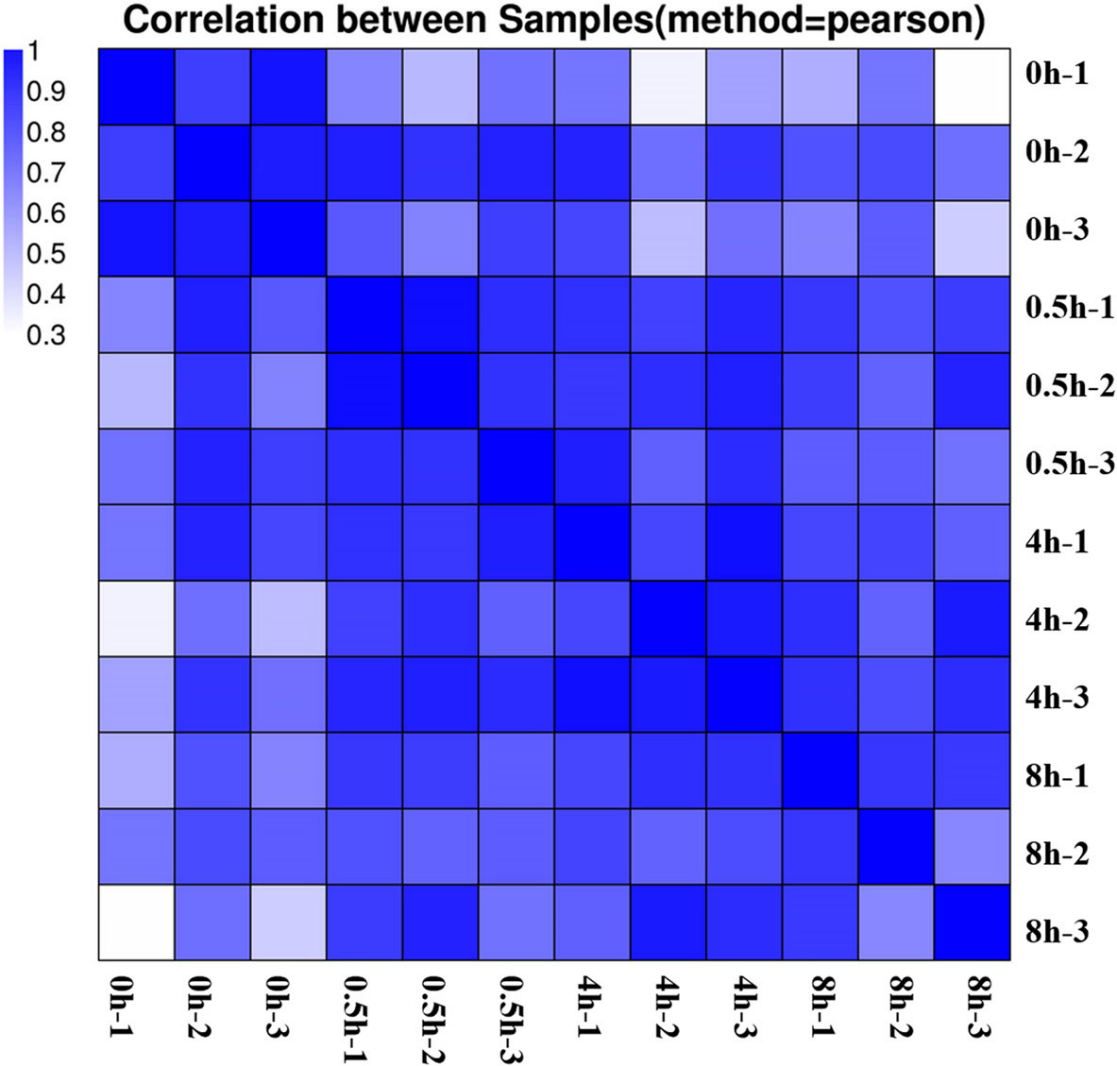
Correlation coefficients for every two samples. Heat map color represents the correlation coefficient; the darker the color, the higher the correlation

In our previous study, RNA-Seq was performed for photosensitive eggplant collected at four time points (0, 0.5, 4 and 8 h after bag removal), and 869 genes were found to be involved in the light-induced anthocyanin biosynthesis [51]. In order to compare the similarities and differences of anthocyanin synthesis in different photosensitive eggplants and to understand the dark-regulated mechanism of anthocyanin synthesis in non-photosensitive eggplant, the same four time points after bag removal were chosen to analyze the transcription of non-photosensitive eggplant. RNA-Seq was performed for three biological replicates at each time point. On average, about 4.45 Gb bases were generated from each sample by Illumina Hiseq platform. After mapping sequenced reads to reference genome and reconstruct transcripts, a total of 27,889 novel transcripts were obtained from all samples. Of these, 14,595 are previously unknown splicing event for known genes, and 1034 are novel coding transcripts without any known features. The remaining 12,260 are long noncoding RNAs. After novel transcript detection, the novel coding transcripts were merged with reference transcript to get the complete reference. The clean reads were then mapped to the reference using Bowtie. The gene expression summary is shown in Additional file 1. To test if the samples chosen were reliable, the correlation value between each two samples was calculated based on FPKM. As shown in Fig. 2, the three replicates at four time points showed a good correlation.

### Analysis of the differentially expressed genes (DEGs)

**Fig. 3:**
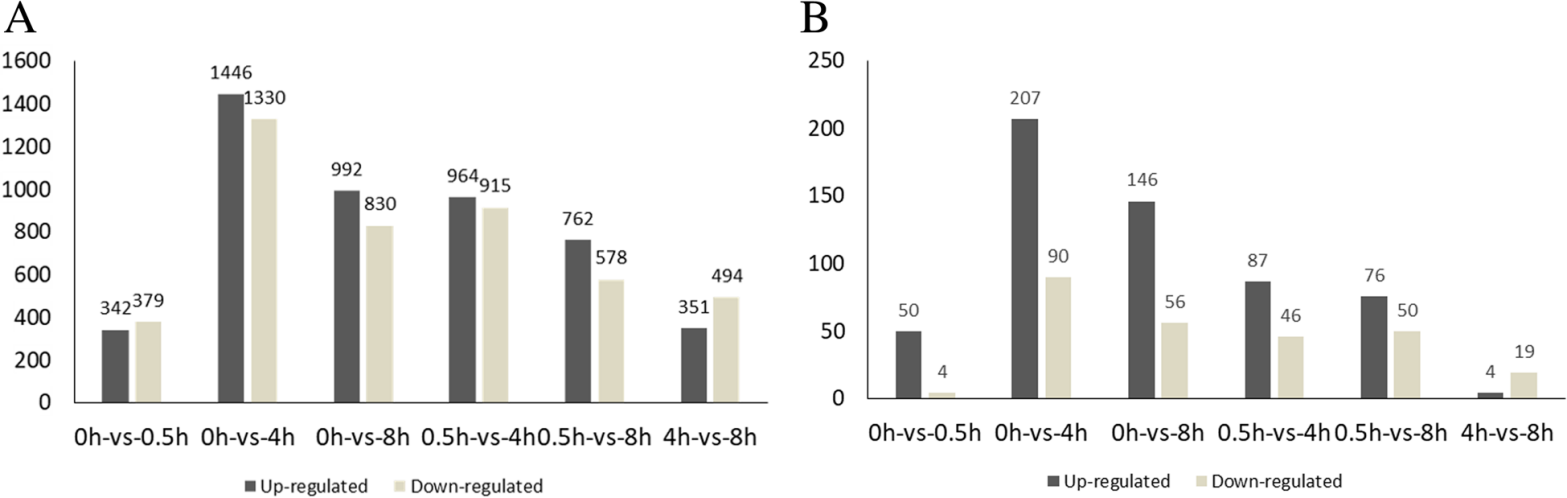
Statistics of DEGs After Opening the Bags. **a** Statistics of up-regulated genes and down-regulated genes at 0 h, 0.5 h, 4 h and 8 h in non-photosensitive eggplant. **b** Statistics of co-up-regulated genes and co-down-regulated genes at 0 h, 0.5 h, 4 h and 8 h in both photosensitive and non-photosensitive eggplant

Three thousand eight hundred forty-one DEGs were identified by comparing FPKM values between different libraries under the thresholds of false discovery rate ≤ 0.001, absolute Log 2 Ratio value ≥1, and divergence probability ≥0.8 (Additional file 2). In non-photosensitive eggplant, 721 DEGs were found at 0.5 h vs 0 h (342 up-regulated, 379 down-regulated), 1879 DEGs at 4 h vs 0.5 h (964 up-regulated, 915 down-regulated), and 845 DEGs at 8 h vs 4 h (351 up-regulated, 494 down-regulated) (Fig. 3a). In photosensitive eggplant, 843 DEGs were found at 0.5 h vs 0 h (703 up-regulated, 140 down-regulated), 865 DEGs at 4 h vs 0.5 h (385 up-regulated, 480 down-regulated), and 223 DEGs at 8 h vs 4 h (65 up-regulated, 158 down-regulated) [51]. By taking the intersection of DEGs of the two materials, there were only 50 co-up-regulated and 4 co-down-regulated DEGs at 0.5 h vs 0 h, 87 co-up-regulated and 46 co-down-regulated DEGs at 4 h vs 0.5 h, and 4 co-up-regulated and 19 co-down-regulated DEGs at 8 h vs 4 h (Fig. 3b). The results indicate that there is much difference between photosensitive and non-photosensitive eggplants in terms of light response patterns and anthocyanin synthesis mechanisms.

### qRT-PCR validation of differentially expressed genes

In order to validate the accuracy of RNA-seq data, *SmMYB1*(Sme2.5_05099.1_g00002.1), *SmMYB73*(Sme2.5_24183.1_g00002.1), *SmTT8*(Sme2.5_00592.1_g00005.1), *SmDFR* (Sme2.5_01401.1_g00004.1), *SmHY5*(Sme2.5_03211.1_g00004.1), *SmWD40*(Sme2.5_05196.1_g00001.1), *SmF3H*(Sme2.5_00015.1_g00020.1), *SmCHI* (Sme2.5_01193.1_g00009.1), *SmbHLH* (Sme2.5_01795.1_g00002.1) and *SmWD40*(Sme2.5_04405.1_g00003.1) related to anthocyanin biosynthesis were selected and subjected to qRT-PCR analysis. The results showed that qRT-PCR highly repeated RNA-seq data and thus verified the accuracy of RNA-seq data (R = 0.78–0.99) **(**Additional file 3**).**

### GO analysis of the DEGs

**Fig. 4:**
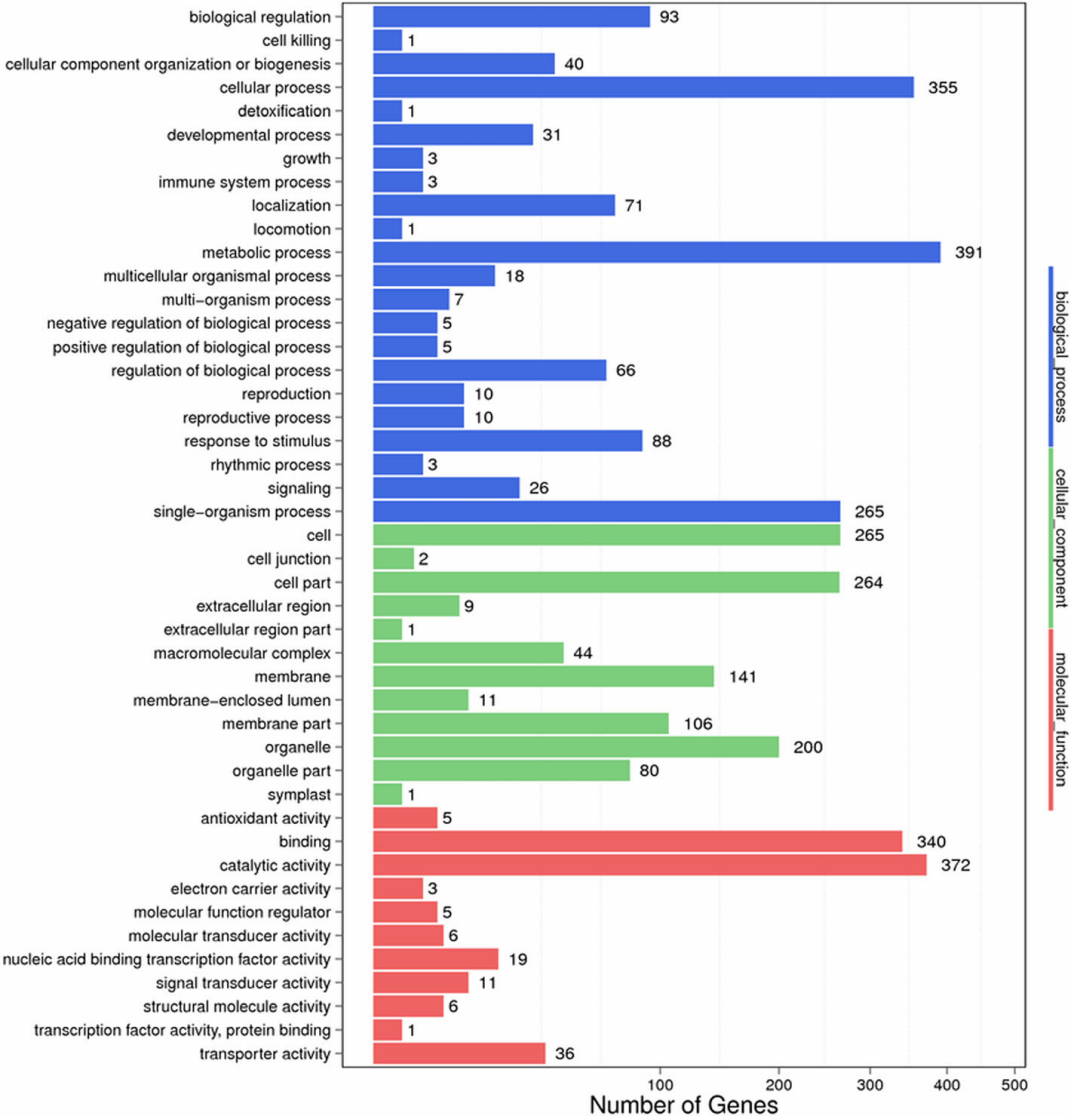
GO classification of DEGs

GO is a useful program for annotation and functional categorization of genes [52]. The GO assignment was used here to classify the functions of the DEGs in eggplant peel. A total of 3841 DEGs were assigned to different GO ontologies based on their sequence similarity to the genes with previously known functions; specifically, 804, 1493, and 1124 DEGs were assigned to the molecular function, the biological process, and the cellular component categories, respectively (Fig. 4). Same as the analysis result for the DEGs in photosensitive eggplant [51], the largest two subcategories in each of the ‘biological process’, the ‘cellular component’, and the ‘molecular function’ categories were ‘metabolic process’ and ‘cellular process’, the ‘cell’ and ‘cell part’, and the ‘catalytic activity’ and ‘binding activity’, respectively.

### Cluster analysis of expression patterns in the DEGs

**Fig. 5:**
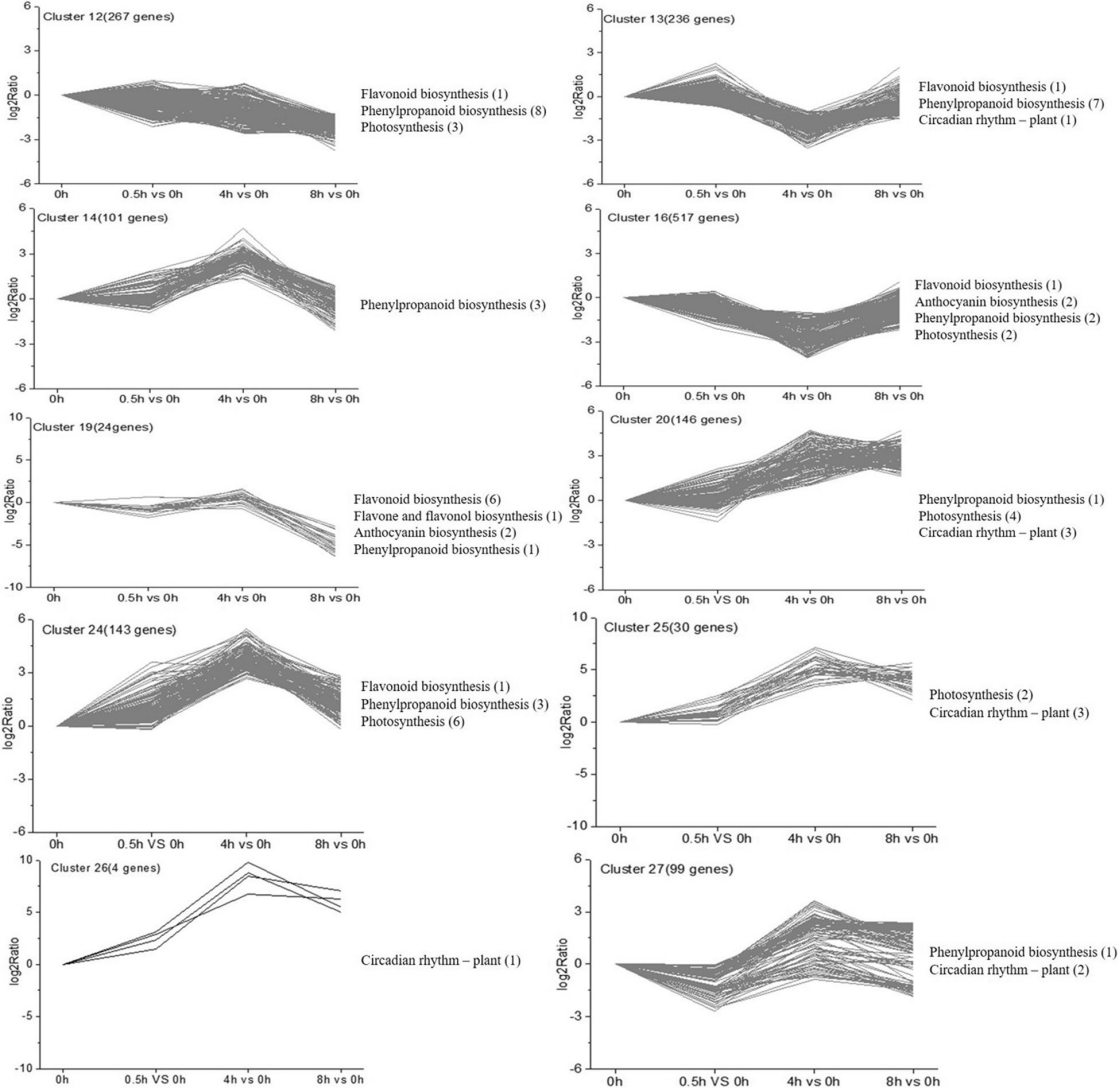
Cluster analysis and KEGG pathway enrichment analysis of DEGs. The x-axis showed the time point comparison. The y-axis shows the relative log2 (ratio) of each comparison. The anthocyanin-related KEGG pathways are listed to the right of each cluster

Three thousand eight hundred forty-one DEGs were subjected to complete-linkage hierarchical clustering using a Euclidean distance metric and divided into 27 clusters (Additional file 4). Genes associated with the anthocyanin biosynthesis were enriched in cluster 12, 13, 14, 16, 19, 20, 24, 25, 26 and 27 (Fig. 5). The expression levels of genes in cluster 12 were down-regulated during 0 h–8 h. The expression levels of genes in clusters 13 and 16 changed slightly at 0.5 h, but were down-regulated at 4 h, and then up-regulated at 8 h. The expression levels of genes in clusters 14, 19, 20, 24, 25, 26, 27 also changed very little at 0.5 h but peaked at 4 h, and were down-regulated at 8 h. Through cluster analysis, it was found that most DEGs related to anthocyanin biosynthesis were down-regulated during the period while in photosensitive eggplant, most such DEGs were up-regulated [51]. This result further illustrates that the regulatory modes for non-photosensitive and photosensitive eggplants were different.

### The structural anthocyanin biosynthesis genes

**Fig. 6:**
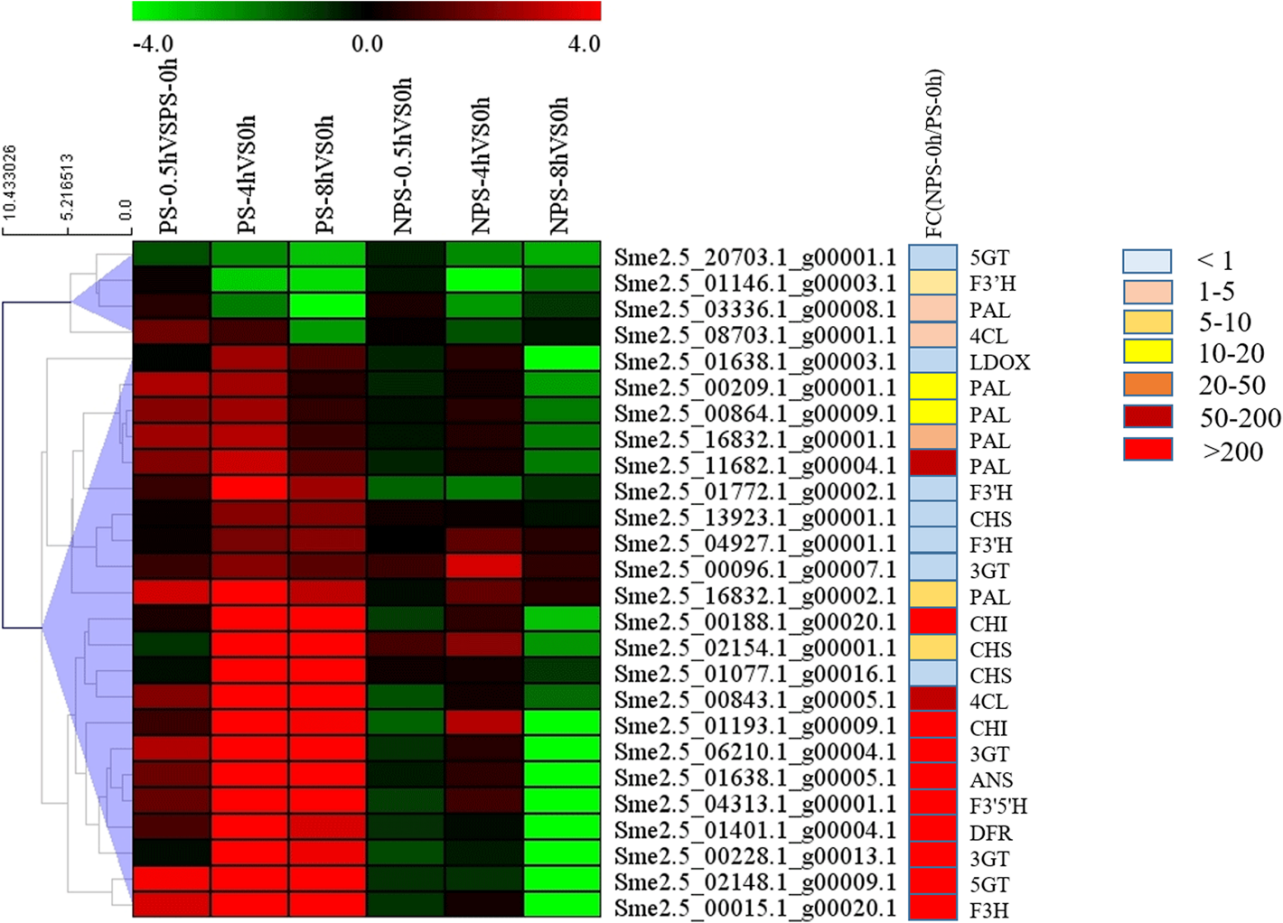
Expression patterns of the structural anthocyanin biosynthesis genes. The color scale represents the Log2 (fold-change to the 0 h time point). Genes that are up-regulated appear in red, and those that are down-regulated appear in green. FC (NPS-0 h / PS-0 h) represents the fold change in gene expression of non-photosensitive and photosensitive eggplants under dark conditions. PS: photosensitive, NPS: non-photosensitive

From the cluster analysis, it was found that the structural anthocyanin biosynthesis genes had different expression patterns for photosensitive and non-photosensitive eggplants. In order to find the key genes for the synthesis of anthocyanin in non-photosensitive eggplants under dark conditions, structural anthocyanin biosynthesis genes with significant changes after opening the bags in photosensitive and non-photosensitive eggplants were merged. As show in Fig. 6, 26 structural anthocyanin biosynthesis genes were divided into two clusters. The genes in cluster 1 were down-regulated after bag removal in both photosensitive and non-photosensitive eggplants, and their expression levels at 0 h were similar to each other, suggesting that these genes did not participate in the anthocyanin synthesis in non-photosensitive eggplant under dark conditions. The genes in cluster 2, most of which had high expression levels in dark conditions in non-photosensitive eggplant, were up-regulated after bag removal in photosensitive eggplant but were down-regulated or unchanged in non-photosensitive eggplant (Fig. 6). The expression levels of *PAL* (Sme2.5_11682.1_g00004.1) and *4CL* (Sme2.5_00843.1_g00005.1) in non-photosensitive eggplant were more than 50 times that in photosensitive eggplant. The expression level of *CHI* (Sme2.5_01193.1_g00009.1, Sme2.5_00188.1_g00020.1), *3GT* (Sme2.5_00228.1_g00013.1, Sme2.5_06210.1_g00004.1), *ANS* (Sme2.5_01638.1_g00005.1), *DFR* (Sme2.5_01401.1_g00004.1), *5GT* (Sme2.5_02148.1_g00009.1), *F3H* (Sme2.5_00015.1_g00020.1) and *F3’5’H* (Sme2.5_04313.1_g00001.1) in non-photosensitive eggplant were more than 200 times that in photosensitive eggplant (Fig. 6). Therefore, it is believed that these genes were involved in the dark-regulated anthocyanin synthesis in non-photosensitive eggplant.

### Light signal perception and transduction

**Fig. 7:**
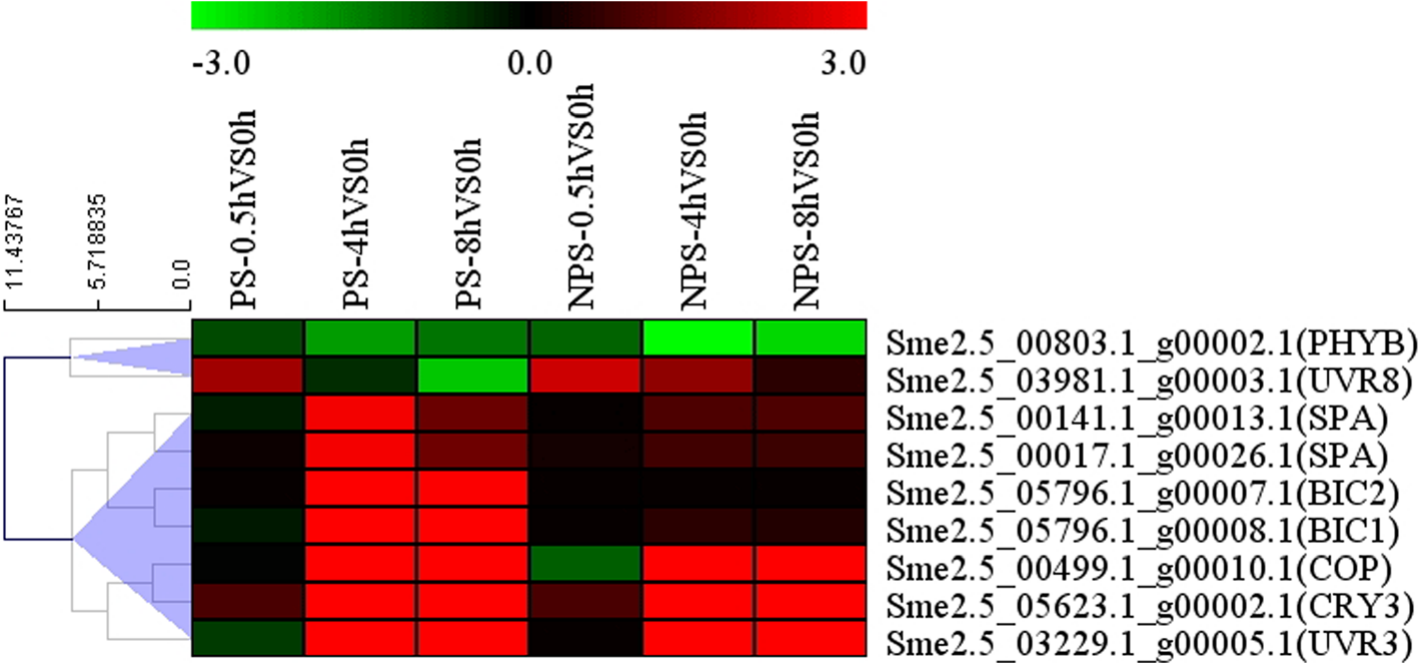
Expression patterns of the photoreceptors and genes related to light signal transduction. The color scale represents the Log2 (fold-change to the 0 h time point). Genes that are up-regulated appear in red, and those that are down-regulated appear in green

In order to clarify the difference between the two eggplants in expression patterns of light signal related genes, the DEGs from photosensitive and non-photosensitive eggplants were combined for comparison. As show in Fig. 7, 9 DEGs were divided into two clusters. In cluster 1, *PHYB* (Sme2.5_00803.1_g00002.1) had the same expression pattern in both photosensitive and non-photosensitive eggplants, but *UVR8* (Sme2.5_03981.1_g00003.1) showed different expression trends in photosensitive and non-photosensitive eggplants after 0.5 h exposure to light. In cluster 2, *COP*, *UVR3* and *CRY3* were up-regulated in both photosensitive and non-photosensitive eggplants after 0.5 h exposure to light; two *SPAs, BIC1 and BIC2,* were up-regulated significantly in photosensitive eggplant, but the up-regulation was insignificant in non-photosensitive eggplant. Thus, we believe that *UVR8*, *SPAs*, *BIC1* and *BIC2* may be involved in dark-regulated anthocyanin biosynthesis in non-photosensitive eggplant.

### Transcription factors related to dark-regulated anthocyanin synthesis

**Fig. 8:**
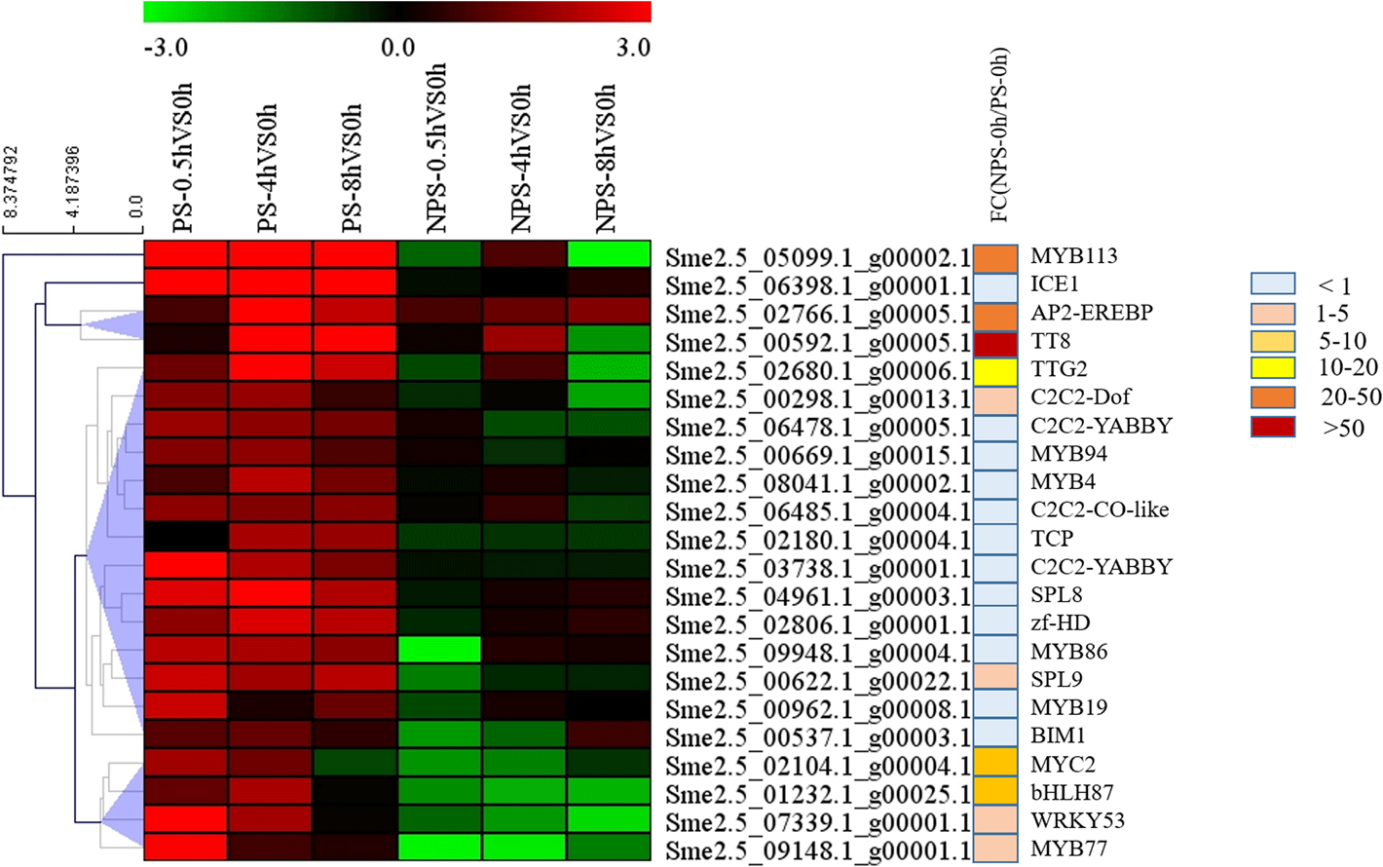
Expression patterns of all the TFs related to dark regulated anthocyanin synthesis. The color scale represents the Log2 (fold-change to the 0 h time point). Genes that are up-regulated appear in red, and those that are down-regulated appear in green. FC (NPS-0 h / PS-0 h) represents the fold change in gene expression of non-photosensitive and photosensitive eggplants under dark conditions

Analysis of the structural and light signal sensing and transduction genes related to anthocyanin biosynthesis showed that those likely involved in dark regulation of anthocyanin synthesis in non-photosensitive eggplant were up-regulated in photosensitive eggplant after bag removal (Fig. 6). However, in non-photosensitive eggplant, the structural genes were down-regulated at 0.5 h and 8 h. Although up-regulated at 4 h, only a few genes’ expressions reached the significant level; the light signal sensing and transduction genes either had the same expression level or had an up-regulated expression trend, but did not reach the significant level at 4 h and 8 h. Since genes with the same expression trend usually have the same function, TFs related to anthocyanin synthesis can be differentiated based on the expression trends of anthocyanin structural genes and light signal sensing and transduction genes. We obtained 338 TFs that changed significantly after removing the bags in photosensitive or non-photosensitive eggplants by merging (Additional file 5); then, we identified 22 TFs that were up-regulated in photosensitive eggplant, but down-regulated or had no significant change in non-photosensitive eggplant (Fig. 8). In the 22 TF genes, *MYB113*, *TT8*, *TTG2* had high expression levels at 0 h in non-photosensitive eggplant. These three TF genes were found to participate in eggplant anthocyanin synthesis in our previous study [15, 53]; *AP2-EREBP*, *MYC2*, *MYB77*, *bHLH87* and *WRKY53* also had high expression level at 0 h in non-photosensitive eggplant, implying their importance to anthocyanin synthesis; Other 14 TF genes were expressed similarly at 0 h in photosensitive and non-photosensitive eggplants. Since one of these TF genes, *SmMYB86*, was considered as a negative regulator of anthocyanin synthesis in our previous studies [51], it is reasonable to believe that other TF genes of the same type may also have participated in dark-regulated anthocyanin synthesis in non-photosensitive eggplant.

### Interaction network construction and target gene prediction of the TFs related to dark regulated anthocyanin synthesis

**Fig. 9:**
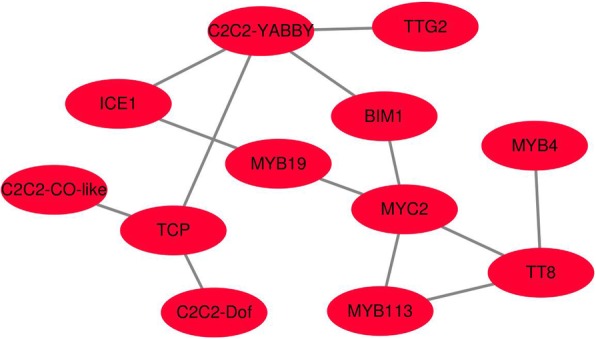
Interaction network of TFs related to dark regulated anthocyanin synthesis

To verify whether the TFs identified in the analysis results were involved in anthocyanin synthesis, we analyzed the 50–1500 bp upstream of the initiation codon of the anthocyanin structural genes and TFs using the I-sanger cloud platform (https://www.i-sanger.com/) for regions that the transcription factors may bind. It was found that these TFs could not only regulate structural genes, but also regulate each other (Additional file 6). Possible interactions among these TFs were also predicted through a protein interaction site (http://netbio.sjtu.edu.cn/arappinet/). As shown in Fig. 9, there were interaction relationships between some of the TFs, and the interaction scores were greater than 0.5 (Additional file 7). These findings suggest that these transcription factors may have the same function and are involved in the dark-regulated anthocyanin biosynthesis in non-photosensitive eggplant.

### Experimental verification of regulatory relationship between dark regulated anthocyanin synthesis related TFs and target genes

**Fig. 10:**
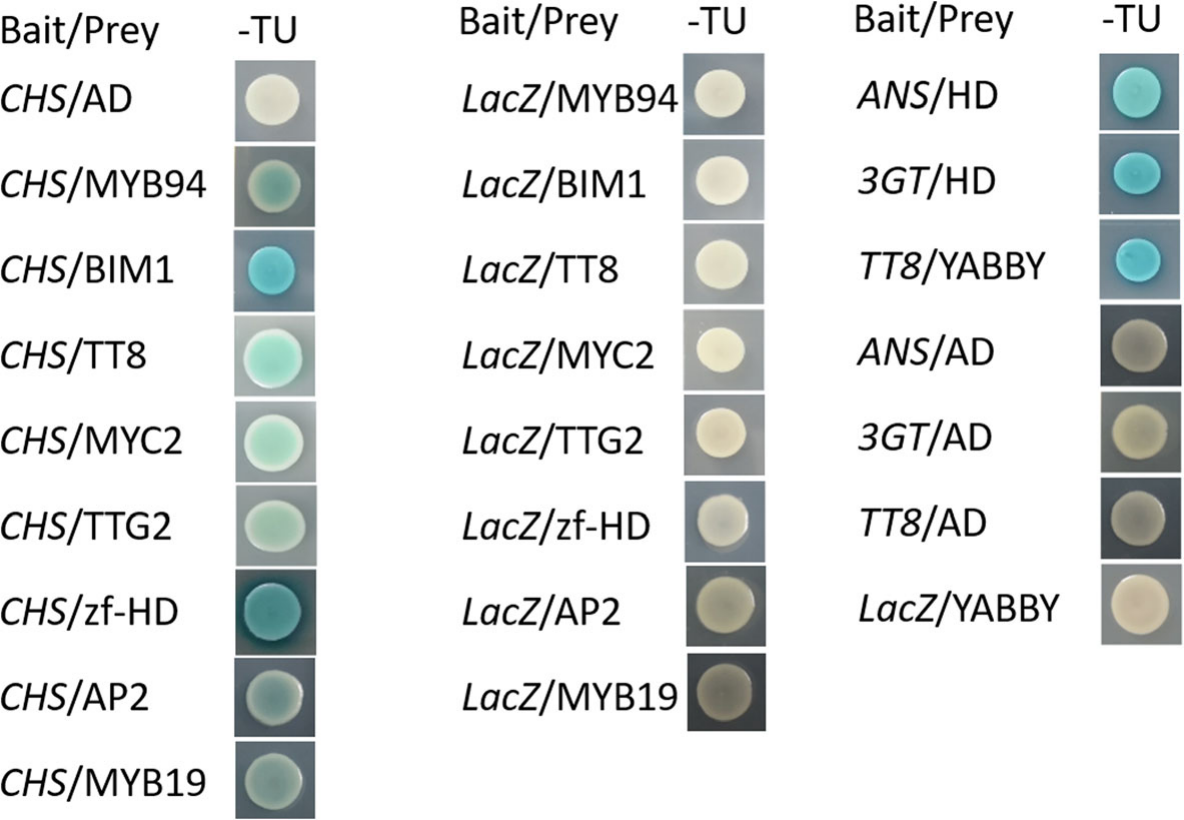
Interactions/bindings between TFs and the promoters of anthocyanin biosynthesis genes, revealed by yeast one-hybrid assays. Indicated combinations of constructs were co-expressed and grown on synthetic dropout –Trp-Ura (−TU). Blue precipitates represented corresponding ß- galactosidase activities

In order to confirm the predicted regulatory relationship between TFs and target genes, the promoters of the non-photosensitive eggplant anthocyanin synthetic structural genes and the coding region of transcription factors were cloned according to target gene prediction results (Additional file 6**).** Through yeast one-hybrid assay, 8 TFs were identified to be direct participants in anthocyanin synthesis, of which SmMYB94, SmBIM1, SmTT8, SmMYC2, SmTTG2, SmAP2, and SmMYB19 can bind to the promoter of *SmCHS* (Sme2.5_13923.1_g00001.1), SmHD can bind to the promoter of *SmCHS* (Sme2.5_13923.1_g00001.1), *SmANS* (Sme2.5_01638.1_g00005.1) and *Sm3GT* (Sme2.5_00228.1_g00013.1), and SmYABBY can bind to the promoter of *SmTT8* (Sme2.5_00592.1_g00005.1) (Fig. 10). Since previous study showed that overexpression of *SmCHS* and *SmANS* can promote anthocyanin synthesis [54]. Thus, TFs that bind to their promoters are likely involved in anthocyanin synthesis.

## Discussion

Our previous study shows that photosensitive eggplant did not synthesize anthocyanins under dark conditions, and anthocyanin synthesis genes and regulatory factors were not expressed; however, the anthocyanin synthesis genes and regulatory factors were significantly induced at 0.5 h, 4 h and 8 h after exposure to light, and anthocyanins were synthesized within a few days [15]. In order to find the key regulators of synthetic anthocyanins in dark conditions to solve the problem of poor coloration of eggplant peel under low light conditions, transcriptome data of photosensitive and non-photosensitive eggplant peel at 0 h, 0.5 h, 4 h, 8 h after illumination were compared and analyzed in this study.

Cluster analysis showed that most of the genes in the phenylpropanoid biosynthesis, flavonoid biosynthesis and anthocyanin biosynthesis pathways were down-regulated in non-photosensitive eggplant after opening bags (Fig. 6). On the other hand, they were up-regulated in photosensitive eggplant [51]. This difference in gene regulation suggests that the mechanisms for anthocyanin biosynthesis in photosensitive and non-photosensitive eggplants were not the same. Through transcriptome comparison, we found that *PAL* (Sme2.5_11682.1_g00004.1), *4CL* (Sme2.5_00843.1_g00005.1), *CHI* (Sme2.5_01193.1_g00009.1, Sme2.5_00188.1_g00020.1), *3GT* (Sme2.5_00228.1_g00013.1, Sme2.5_06210.1_g00004.1), *ANS* (Sme2.5_01638.1_g00005.1), *DFR* (Sme2.5_01401.1_g00004.1), *5GT* (Sme2.5_02148.1_g00009.1), *F3H* (Sme2.5_00015.1_g00020.1) and *F3’5’H* (Sme2.5_04313.1_g00001.1) were up-regulated in photosensitive eggplant, but down-regulated in non-photosensitive eggplant after bags removal, and they have a high expression level in dark conditions in non-photosensitive eggplant (Fig. 6). Many studies demonstrated that overexpression or antisense of these structural genes resulted in changes in anthocyanin accumulation. Compared to untransformed lines, overexpression of *CHS* gene from *Malus crabapple* in tobacco resulted in anthocyanin accumulation [55]. In *Malus domestica*, *chs* knockout lines had no detectable anthocyanins and had radically reduced concentrations of flavonoids [56]. Overexpression of the *CHI* gene of *petunia* in tomato (*Solanum lycopersicum*) resulted in flavonol accumulation in fruit peel [57]. *F3H*-silenced strawberry fruit displayed reduced anthocyanins and flavonol content [58]. Overexpressing *PtrDFR1* in Chinese white poplar (*P. tomentosa Carr.*) increased the accumulation of anthocyanins and condensed tannins [59]. Silencing *DFR* expression in sweet potato (*Dioscorea esculenta*) significantly decreased anthocyanin content in storage roots and stems [60]. Overexpression of *CHS*, *CHI*, *F3H* and *DFR* of *Solanum melongena* in *Arabidopsis thaliana* successfully increased the pigmentation in the stems and siliques [54]. The anthocyanin content was significantly lower in the *Pe3GT*-suppressed *Phalaenopsis* flowers [61]. Taken as a whole, both the anthocyanin accumulation and pigmentation phenotype were in agreement with the above results.

Structural genes of anthocyanin biosynthesis are simultaneously regulated by the activated TFs. In this study, through comparative transcriptiome we also found 22 TFs that have the same expression pattern as the structural genes (Fig. 8). The 22 TFs can be divided into 8 categories which contained six MYBs, five bHLHs, two WRKYs, four C2C2s, two SPLs, HD, TCP, and AP2. Among these TFs, three MYBs (MYB113, MYB94 and MYB19), three bHLHs (TT8, MYC2 and BIM1), WRKY (TTG2), HD, and AP2 can bind directly to the promoter of *CHS* (Sme2.5_13923.1_g00001.1), and one C2C2 (YABBY) can bind to the promoter of *TT8* (Fig. 10). Although other transcription factors can not bind directly to the promoters of the structural genes, they were predicted to be involved in anthocyanin synthesis by binding to the promoter of TFs which can bind directly to the promoter of anthocyanin structural genes (Additional file 6). Studies also showed that *MYB113* (*MYB1* or *MYB75*) [30, 33, 62, 63], *TT8* [40, 64, 65] and *TTG2* (*WRKY44*, [66, 67]) were involved in anthocyanin synthesis in many plant species. In addition, other TFs such as SPL [47], TCP (TCP3 and TCP15, [45, 46], AP2 [44], WRKY (WRKY41 and WRKY11, [68, 69]) and MYC (MYC1, [29]) were also characterized to be involved in anthocyanin synthesis. At present, there is no report on the involvement of homeodomain-like protein (HD) and C2C2 type zinc finger protein in the synthesis of anthocyanins and flavonoids. In this study, *MYB113*, *TT8*, *TTG2*, *MYC2* and *AP2* have a high expression level in dark condition in non-photosensitive eggplant, and the expression levels of *MYB94*, *MYB19*, *BIM1* and *HD* were not much different between the two eggplants in dark condition, but they have different expression patterns in the two materials. Since these TFs bind directly to the promoter of *CHS* (Sme2.5_13923.1_g00001.1), they were thought to have participated in anthocyanin biosynthesis in eggplant.

While light is one of the key environmental factors for the anthocyanin synthesis in eggplant, this study found that anthocyanins can still proceed without light. Transcriptome comparison analysis showed that *UVR*8 has different expression pattern among the two types of eggplants (Fig. 7). It has also been shown that *UVR8* can promote anthocyanin synthesis in radish sprouts and Arabidopsis, but the synthesis was dependent of light [70, 71]. It was reported that AtUVR8 can interact directly with AtBIM1 and AtWRKY36 to participate in hypocotyl elongation [72, 73]. We also confirmed in this study that SmBIM1 and SmWRKY53 can directly bind to the promoter of *SmCHS* (Sme2.5_13923.1_g00001.1). Based on these results, we hypothesize that SmUVR8 can directly interact with SmWRKY53 and SmBIM1 to regulate eggplant anthocyanin synthesis. COP1, an E3 ubiquitin ligase, can form a complex with SPA to regulate anthocyanin synthesis of Arabidopsis; *cop1* and *spa* mutants can produce anthocyanins also in the dark [26]. Jiang et al. reported that SmCOP1 (Sme2.5_00128.1_g00013.1) can interact with SmHY5 and SmMYB113 to regulate anthocyanin biosynthesis and restore the phenotype of the Arabidopsis *cop* mutant [15]. In this study, we found two SPAs and COP1 (Sme2.5_00499.1_g00010.1) which had different expression patterns in the two materials and thus might have participated in the dark-regulated anthocyanin synthesis in non-photosensitive eggplant (Fig. 7). Cryptochromes are blue-light receptors that regulate plant development and the circadian clock. CRY2 undergoes blue light–dependent homodimerization to become physiologically active. The binding of BICs to CRY2 inhibits the blue light–dependent dimerization, thereby regulating cell photosensitivity [16, 74]. In this study, we found that *BIC* and *BIC2* were up-regulated in photosensitive eggplant after bag removal; yet, there was essentially no expression of these genes in non-photosensitive eggplant, leading us to suspect that the non-photosensitive eggplant peel cells might have lost photosensitivity in some cases.

## Conclusions

In this study, comparative transcriptome was applied to elucidate the underlying molecular mechanism for dark-regulated anthocyanin biosynthesis in non-photosensitive eggplant. The analyses revealed that the structural genes and the TFs involved in anthocyanin synthesis were down-regulated after bag removal, while most of them have high expression levels in dark conditions in non-photosensitive eggplant. Through transcription factor target gene prediction and yeast one-hybrid verification, we believe that *SmBIM1*, *SmHD*, *SmMYB94*, *SmMYB19*, *SmTT8*, *SmYABBY*, *SmTTG2*, *SmAP2* and *SmMYC2* may be involved in dark-regulated anthocyanin synthesis in non-photosensitive eggplant. Our results shaded some lights on the understanding of molecular mechanism of dark-regulated anthocyanin synthesis in non-photosensitive eggplant.

## Methods

### Plant materials

The non-photosensitive eggplant material ‘145’, obtained through a large number of bagging screening of fruits with different eggplant genotypes, was provided by the Shanghai Academy of Agricultural Sciences, Shanghai, China. ‘145’ was grown in the greenhouse of Shanghai Jiao Tong University, Shanghai, China, under natural light conditions. For fruit bagging treatment, sepals were covered with double-layer Kraft paper bags after full bloom. After the bagging of the materials for 2 weeks, the bags were opened to collect materials at 8:00 am. Three materials were selected as biological replicates, with each individual material being sampled from every bagged plant at 0 h, 0.5 h, 4 h, and 8 h after the bags were removed. All peel samples were frozen immediately with liquid nitrogen and kept at − 80 °C.

### Anthocyanin content determination

Anthocyanin content was measured with the pH differential spectrophotometry method [75]. 0.5 g materials were used to extract anthocyanin with 0.01% HCl in 0.5 ml methanol at 4 °C for 12 h. The extracts were centrifuged at 8500 g for 20 min. The supernatant was transferred to a 10 ml tube and the sediments were further processed with 5 ml extraction solution at 4 °C for 6 h. The extracts from the sediments were also centrifuged at 8500 g for 20 min. Spectrophotometrical absorbance was measured at 510 nm and at 700 nm in buffers at pH 1.0 and pH 4.5. The anthocyanin content was calculated according to the following formula: TA = A × MW × 5 × 100 × V/ε, where TA stands for total anthocyanin content (mg/100 g), V is the final volume (ml), A equals to [A510 nm (pH 1.0) − A700 nm (pH 1.0)] − [A510 nm (pH 4.5) − A700 nm (pH 4.5)], ε is the molar absorptivity which is 26,900, MW is the molecular weight with a value of 449.2, and A510 and A700 represent the absorbance values at 510 and 700 nm, respectively [76]. Three measurements were performed for each sample replicate.

### RNA extraction, cDNA library construction, and RNA-Seq

Total RNAs were extracted from pericarp using the RNAiso plus kit (TaKaRa, Otsu, Shiga, Japan) and following the manufacturer’s instructions therein. RNase-free DNase (Takara, Japan) was used to remove the potential genomic DNAs in extracted RNAs. After total RNAs were extracted and treated with DNase I, Oligo (dT) was used to isolate mRNA. The mRNAs were fragmented through mixing with the fragmentation buffer. cDNA was then synthesized using the mRNA fragments as templates. Short fragments were purified and resolved with EB buffer for end reparation and single nucleotide A (adenine) addition. The short fragments were then connected with adapters. Suitable fragments were selected for the PCR amplification. The quality and quantity of RNAs were determined by Agilent 2100 Bioanalyzer and ABI StepOne Plus Real-Time PC System. Finally, the library was sequenced using Illumina HiSeq™ 2000.

### Sequence assembly and gene annotation

Low-quality, adaptor-pollute, and high content of unknown base (N) reads were removed by QC before downstream analyses. After reads filtering, the clean reads were mapped to the reference genome by HISAT. Eggplant reference genome database (http://vegmarks.nivot.affrc.go.jp/VegMarks/jsp/index.jsp) was used as the reference. After the novel transcripts were obtained, their coding transcripts were merged with reference transcripts to get the complete reference which was used for gene expression analysis.

### Identification and annotation of DEGs

After the setup of the complete reference, the clean reads were mapped to the reference library using Bowtie2; then gene expression level for each sample was calculated by RSEM. DEGs were detected by DEseq2 and fold change ≥ 2.00 and adjusted *p*-value ≤ 0.05 were taken as conditions. Gene Ontology (GO) classification and functional enrichment were performed by WEGO software (http://wego.genomics.org.cn/cgi-bin/wego/index.pl). KEGG pathway classification and functional enrichment for DEGs were performed by the database-Kyoto Encyclopedia of Genes and Genomes. Cluster analysis of expression patterns were analyzed by MeV (4.9).

### Real-time qPCR analysis

Real-time quantitative reverse transcription-PCR was used to estimate the accuracy of RNA-Seq. Total RNA was treated with DNase to remove trace of DNA. 1 μg RNA was used for synthesis of cDNA with the PrimeScript RT Master Mix Perfect Real Time Kit (Takara). Quantitative real-time RT-PCR (qPCR) was performed on FTC-3000 real-time PCR System (Funglyn Biotech, Canada) using the following program: 95 °C for 30 s, followed by 40 cycles of 95 °C for 20 s, 58 °C for 30 s, and 72 °C for 30 s, which was consistent with the manufacturer’s instructions of SYBR Premix Ex Taq II Kit (Takara). The Actin gene (GU984779.1) was used as an internal reference, and relative expression was calculated using the 2ΔCt method. Each qPCR analysis was performed in triplicate. The relative expression levels of the amplified products were analyzed using the comparative CT method based on CT values. All primers used in this study are listed in Additional file 8.

### Yeast one-hybrid assay

The procedure for the yeast one-hybrid assay was described in a previous report [34]. 50–1500 bp upstream of the initiation codon of the anthocyanin structural genes was amplified from non-photosensitive eggplant and fused to the vector LacZ. The coding regions of the transcription factors were amplified from non-photosensitive eggplant and fused to the vector PB4 2 AD. The constructs were co-transformed into the yeast strain EGY48 in the indicated combinations. After 3 days of transformation, 6 yeast clones grown on the medium-synthesized sputum-Trp-Ura (SD-TU) were selected and cultured in liquid SD-TU medium for 24 h, and the culture was inoculated to contain 80 mg/ L X-gal in SD-TU medium.

## Additional files

Additional file 1: **Table S2.** Gene Expression Summary. (DOCX 15 kb)

Additional file 2: **Table S3.** Differentially expressed genes (DEGs) in non-photosensitive eggplant. (XLSX 239 kb)

Additional file 3: **Figure S1.** qRT-PCR Analysis of DEGs after Opening the Bags. (DOCX 522 kb)

Additional file 4: **Figure S2.** Cluster analysis and KEGG pathway enrichment analysis of DEGs. (DOCX 1176 kb)

Additional file 5: **Table S4.** Differentially expressed TF genes in non-photosensitive and photosensitive eggplant. (XLSX 45 kb)

Additional file 6: **Table S5.** Transcription factors target gene prediction results. (DOCX 35 kb)

Additional file 7: **Table S6.** The interaction score of transcription factors in Fig. 9. (DOCX 13 kb)

Additional file 8: **Table S1.** List of all the primer sequences used in this study. (DOCX 15 kb)

## Data Availability

The Illumina RNA-seq data generated from non-photosensitive eggplant are available in the NCBI SRA with accessions number SRA613313, and the RNA-seq data of photosensitive eggplant are available in the NCBI SRA with accessions numbers SRR5650714, SRR5651526, SRR5658205 and SRR5658226 [51].

## Abbreviations

4CL: 4-coumarate-CoA ligase
ANS: Anthocyanin synthase
AT: Acylation
CHI: Chalcone isomerase
CHS: Chalcone synthase
COL: CONSTANS-like
COP1: Constitutive Photomorphogenic 1
CRY3: Cryptochrome 3
DEGs: Differentially expressed genes
DFR: Dihydroflavonol reductase
F3H: Flavanone 3-hydroxyl enzyme
FDR: False discovery rate
GO: Gene ontology
GT: Glycosylation
HFR: Hypocotyl in Far-Red 1
HY5: Hypocotyl 5
HYH: Hypocotyl homolog
KEGG: Kyoto encyclopedia of genes and genomes
LDOX: Leucoanthocyanidin dioxygenase
MT: Methylation
NPS: non-photosensitive
Nr: NCBI non-redundant database
PAL: Phenylalanine ammonia lyase
PHOTs: Phototropins
PHYs: Phytochromes
PS: photosensitive
QC: Quality control
SPL9: Squamosa promoter binding protein-like 9
SRA: Sequence read archive
UVR8: UV Resistance Locus 8
