# Supplementary material for: Comparative transcription analysis of photosensitive and non-photosensitive eggplants to identify genes involved in dark regulated anthocyanin synthesis
Source: BMC Genomics. 2019 Aug 28;20:678. doi: 10.1186/s12864-019-6023-4 (PMC6712802; doi:10.1186/s12864-019-6023-4)
Supplement: Supplementary file 1 — Table S2. Gene Expression Summary. (DOCX 15 kb) [file 12864_2019_6023_MOESM1_ESM.docx]

| Sample | Total Clean Reads | Total MappingRatio | Uniquely MappingRatio | Total Gene Number | Novel Gene Number | Total Transcript Number | Novel Transcript Number |
| --- | --- | --- | --- | --- | --- | --- | --- |
| 0H-1 | 28151890 | 81.35% | 77.26% | 24022 | 905 | 30626 | 12689 |
| 0H-2 | 30578742 | 80.34% | 76.35% | 23019 | 899 | 29798 | 12839 |
| 0H-3 | 25732730 | 81.93% | 78.10% | 22244 | 906 | 29060 | 12687 |
| 0.5H-1 | 29559376 | 81.83% | 78.08% | 21678 | 892 | 28120 | 12595 |
| 0.5H-2 | 29361012 | 82.22% | 78.06% | 19712 | 899 | 25891 | 12475 |
| 0.5H-3 | 30589824 | 81.13% | 76.52% | 21003 | 920 | 27559 | 12717 |
| 4H-1 | 30744392 | 80.62% | 76.16% | 21440 | 934 | 28340 | 12959 |
| 4H-2 | 30563862 | 80.45% | 76.60% | 20984 | 893 | 27477 | 12368 |
| 4H-3 | 30695392 | 81.05% | 76.54% | 21524 | 904 | 28318 | 12761 |
| 8H-1 | 30162778 | 78.47% | 75.66% | 22074 | 955 | 29365 | 13358 |
| 8H-2 | 29411208 | 81.82% | 78.66% | 22803 | 942 | 30118 | 13349 |
| 8H-3 | 30380236 | 78.02% | 74.42% | 20232 | 891 | 26370 | 12176 |

**Table S2 Gene Expression Summary**
