# Supplementary material for: Comparative transcription analysis of photosensitive and non-photosensitive eggplants to identify genes involved in dark regulated anthocyanin synthesis
Source: BMC Genomics. 2019 Aug 28;20:678. doi: 10.1186/s12864-019-6023-4 (PMC6712802; doi:10.1186/s12864-019-6023-4)
Supplement: Supplementary file 3 — Figure S1. qRT-PCR Analysis of DEGs after Opening the Bags. (DOCX 522 kb) [file 12864_2019_6023_MOESM3_ESM.docx]

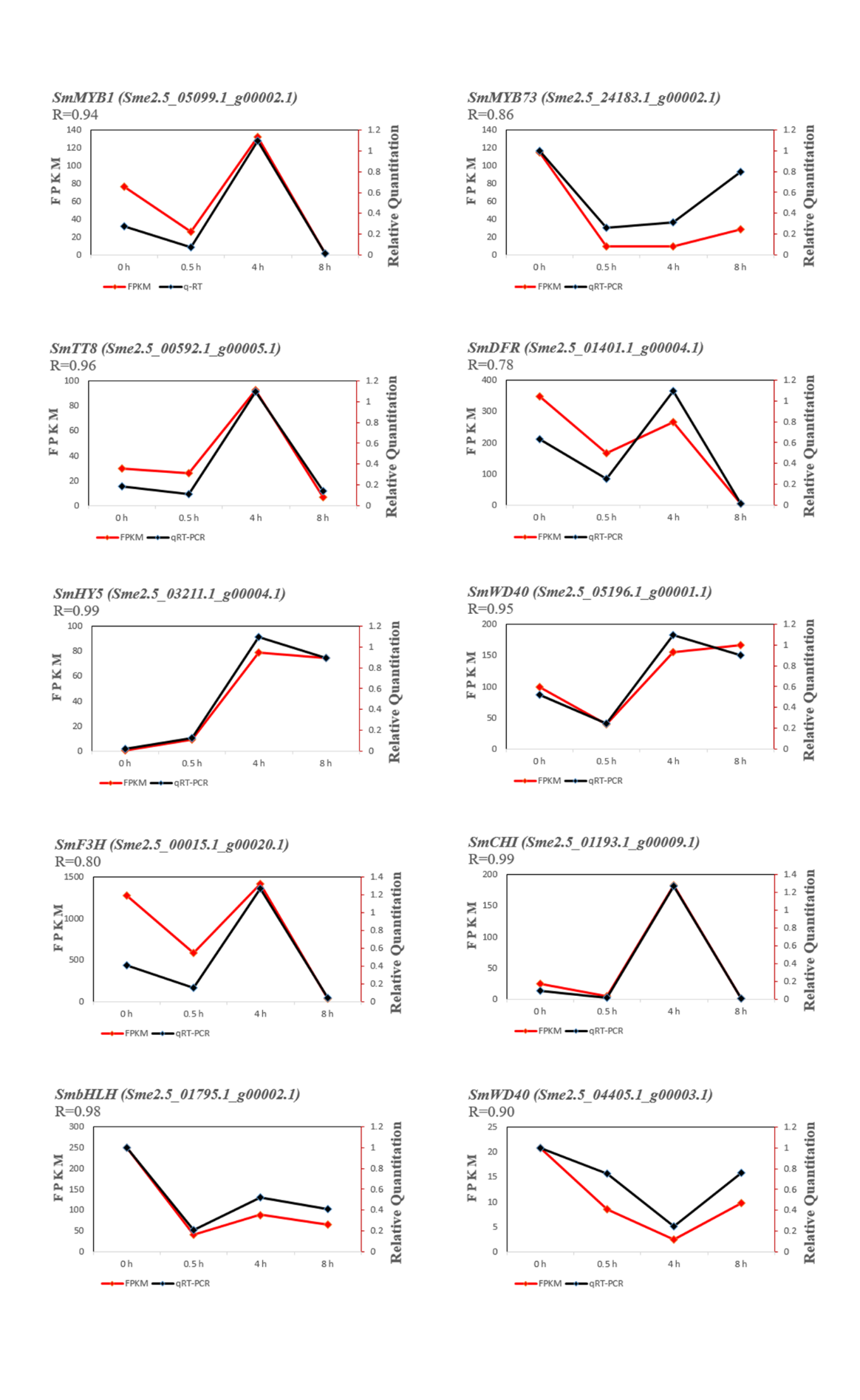


**Figure S1 qRT-PCR Analysis of DEGs after Opening the Bags**

The black line represents FPKM (Fregments Per Kilobase per Million) and the red line represents the expression levels analyzed by qRT-PCR; pearson correlation coefficients R range between 0.78 and 0.99 and averaged 0.915.
