# Supplementary material for: Comparative transcription analysis of photosensitive and non-photosensitive eggplants to identify genes involved in dark regulated anthocyanin synthesis
Source: BMC Genomics. 2019 Aug 28;20:678. doi: 10.1186/s12864-019-6023-4 (PMC6712802; doi:10.1186/s12864-019-6023-4)
Supplement: Supplementary file 4 — Figure S2. Cluster analysis and KEGG pathway enrichment analysis of DEGs. (DOCX 1176 kb) [file 12864_2019_6023_MOESM4_ESM.docx]

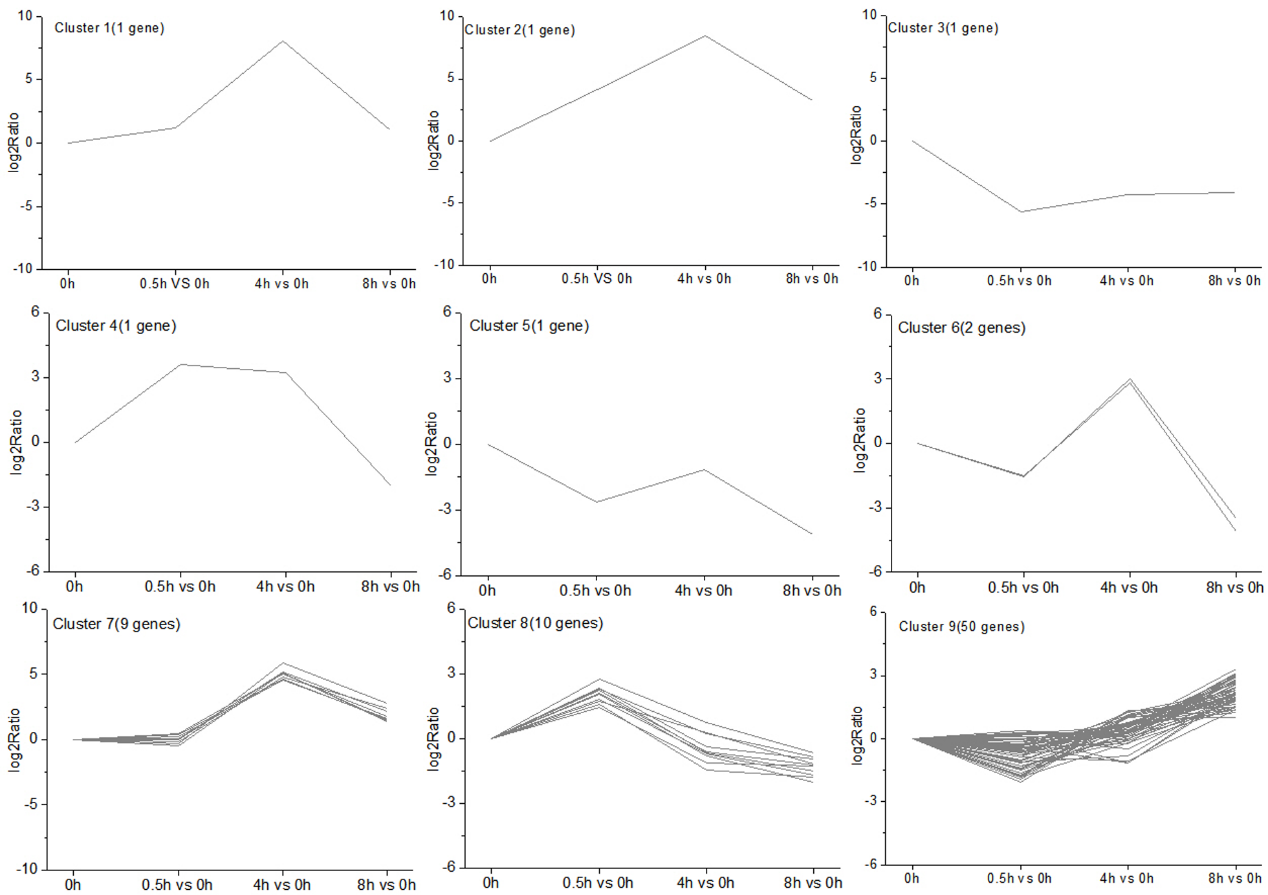


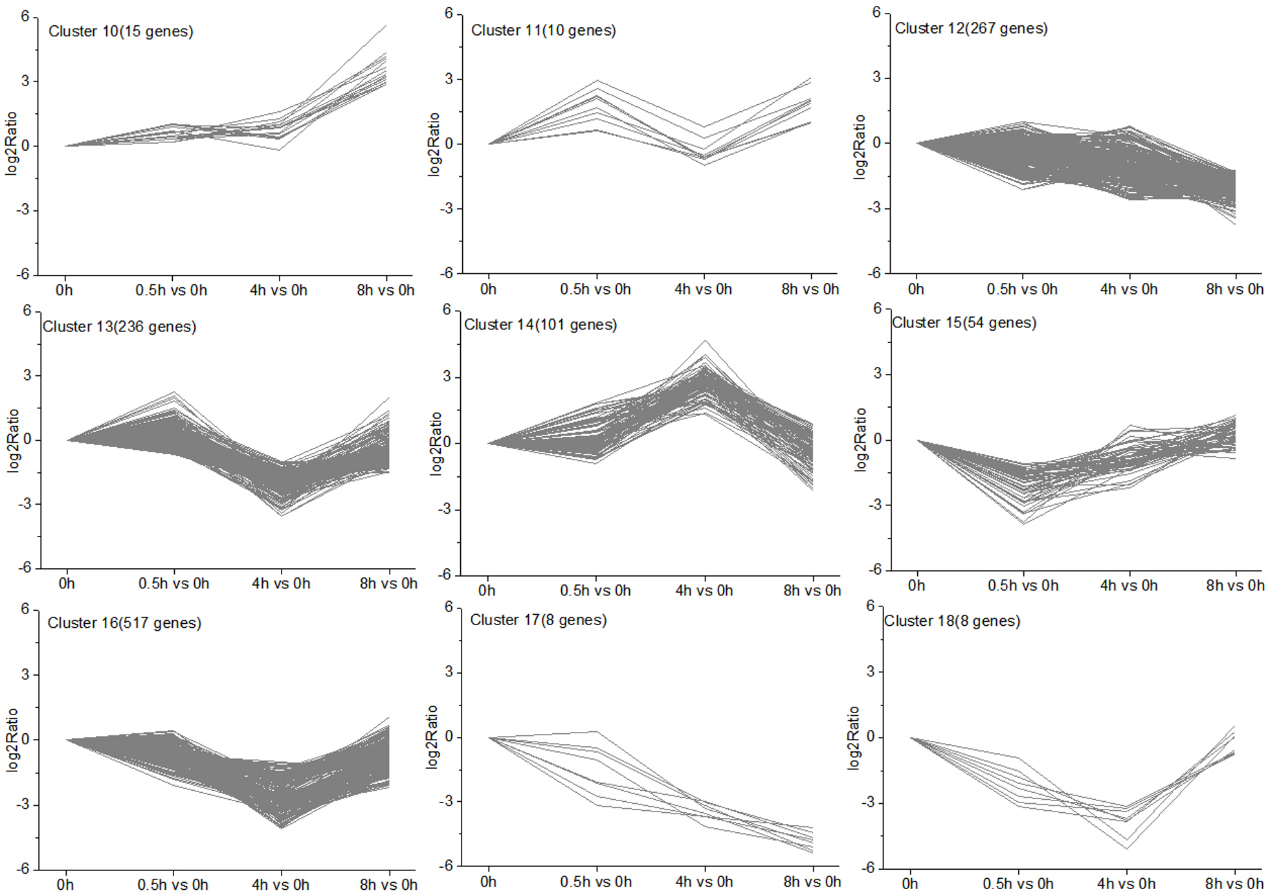


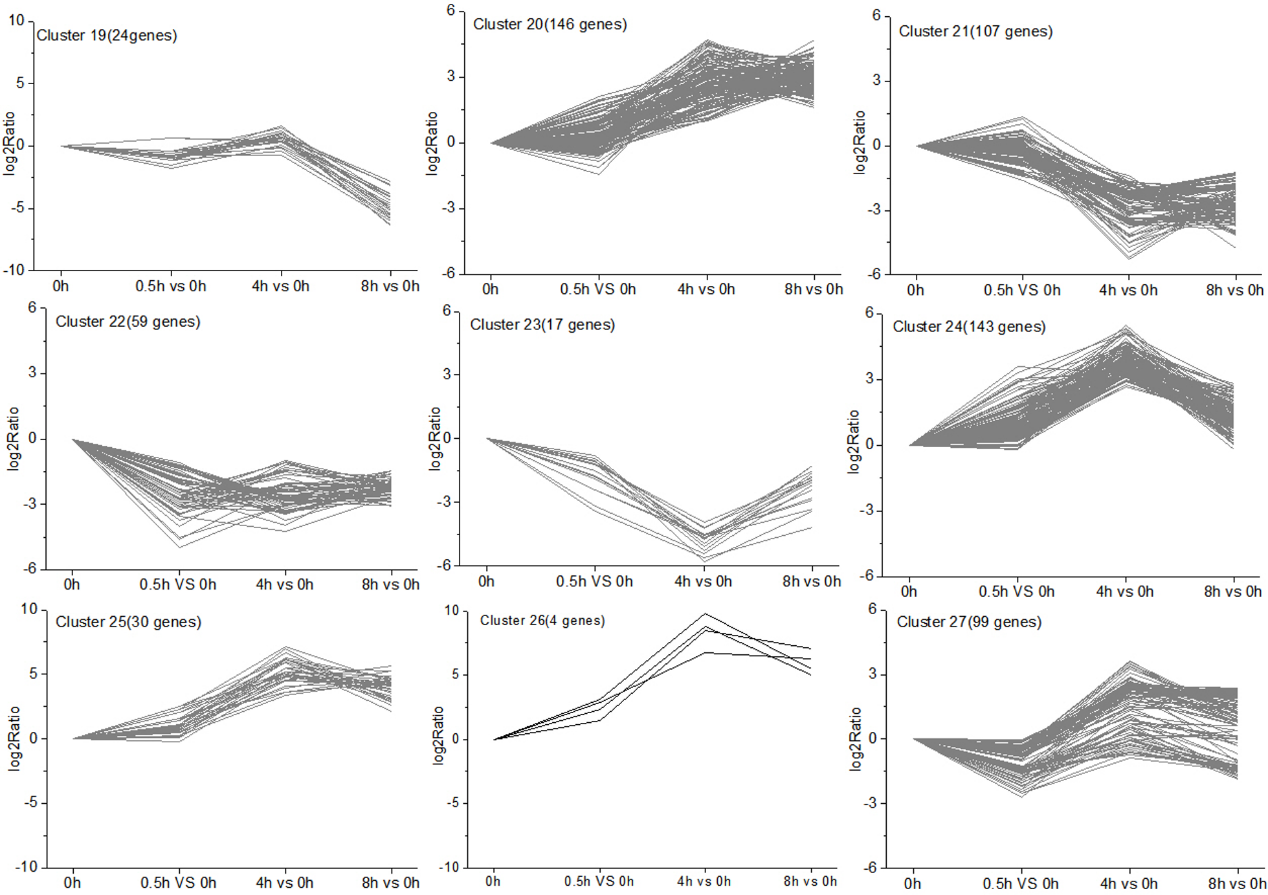


**Figure S2 Cluster analysis and KEGG pathway enrichment analysis of DEGs. The x-axis showed the time point comparison. The y-axis shows the relative log2 (ratio) of each comparison.**
