# Supplementary material for: Comparative transcription analysis of photosensitive and non-photosensitive eggplants to identify genes involved in dark regulated anthocyanin synthesis
Source: BMC Genomics. 2019 Aug 28;20:678. doi: 10.1186/s12864-019-6023-4 (PMC6712802; doi:10.1186/s12864-019-6023-4)
Supplement: Supplementary file 6 — Table S5. Transcription factors target gene prediction results. (DOCX 35 kb) [file 12864_2019_6023_MOESM6_ESM.docx]

Table S5 Transcription factors target gene prediction results

| TF id | TF name | Motif_id | Target_gene_id | target name | p-value | q-value | start | stop | matched_sequence |
| --- | --- | --- | --- | --- | --- | --- | --- | --- | --- |
| Sme2.5_05099.1_g00002.1 | MYB113 | MP00213 | Sme2.5_00228.1_g00013.1 | 3GT | 9.07E-05 | 0.433 | 486 | 496 | TTGTTCGTTAT |
| Sme2.5_05099.1_g00002.1 | MYB113 | MP00213 | Sme2.5_02148.1_g00009.1 | 5GT | 5.02E-05 | 0.433 | 1369 | 1379 | AGGTCAGTTAC |
| Sme2.5_05099.1_g00002.1 | MYB113 | MP00213 | Sme2.5_01193.1_g00009.1 | CHI | 8.08E-05 | 0.433 | 1119 | 1129 | AAATTAGTTAT |
| Sme2.5_05099.1_g00002.1 | MYB113 | MP00213 | Sme2.5_01193.1_g00009.1 | CHI | 8.08E-05 | 0.433 | 1131 | 1141 | AAATTAGTTAT |
| Sme2.5_05099.1_g00002.1 | MYB113 | MP00213 | Sme2.5_13923.1_g00001.1 | CHS | 1.99E-05 | 0.416 | 521 | 531 | AATTCAGTTAT |
| Sme2.5_05099.1_g00002.1 | MYB113 | MP00213 | Sme2.5_04313.1_g00001.1 | F3'5'H | 1.08E-05 | 0.416 | 274 | 284 | AAGTTAGTTAC |
| Sme2.5_03738.1_g00001.1 | C2C2-YABBY | MP00620 | Sme2.5_00228.1_g00013.1 | 3GT | 4.98E-05 | 0.0691 | 1060 | 1069 | CAATAATAAC |
| Sme2.5_03738.1_g00001.1 | C2C2-YABBY | MP00620 | Sme2.5_00228.1_g00013.1 | 3GT | 5.18E-05 | 0.0691 | 10 | 19 | CAATCATCAT |
| Sme2.5_03738.1_g00001.1 | C2C2-YABBY | MP00620 | Sme2.5_00228.1_g00013.1 | 3GT | 5.18E-05 | 0.0691 | 79 | 88 | CAATCATCAT |
| Sme2.5_03738.1_g00001.1 | C2C2-YABBY | MP00620 | Sme2.5_00228.1_g00013.1 | 3GT | 5.18E-05 | 0.0691 | 148 | 157 | CAATCATCAT |
| Sme2.5_03738.1_g00001.1 | C2C2-YABBY | MP00620 | Sme2.5_00228.1_g00013.1 | 3GT | 5.18E-05 | 0.0691 | 208 | 217 | CAATCATCAT |
| Sme2.5_03738.1_g00001.1 | C2C2-YABBY | MP00620 | Sme2.5_00228.1_g00013.1 | 3GT | 5.18E-05 | 0.0691 | 277 | 286 | CAATCATCAT |
| Sme2.5_03738.1_g00001.1 | C2C2-YABBY | MP00620 | Sme2.5_00228.1_g00013.1 | 3GT | 5.18E-05 | 0.0691 | 346 | 355 | CAATCATCAT |
| Sme2.5_03738.1_g00001.1 | C2C2-YABBY | MP00620 | Sme2.5_00228.1_g00013.1 | 3GT | 5.18E-05 | 0.0691 | 406 | 415 | CAATCATCAT |
| Sme2.5_03738.1_g00001.1 | C2C2-YABBY | MP00620 | Sme2.5_00228.1_g00013.1 | 3GT | 9.44E-05 | 0.0766 | 1061 | 1070 | TAATAATAAT |
| Sme2.5_03738.1_g00001.1 | C2C2-YABBY | MP00620 | Sme2.5_00843.1_g00005.1 | 4CL | 4.98E-05 | 0.0691 | 1212 | 1221 | CAATAATAAC |
| Sme2.5_03738.1_g00001.1 | C2C2-YABBY | MP00620 | Sme2.5_02148.1_g00009.1 | 5GT | 5.18E-05 | 0.0691 | 919 | 928 | CAATCATCAT |
| Sme2.5_03738.1_g00001.1 | C2C2-YABBY | MP00620 | Sme2.5_02148.1_g00009.1 | 5GT | 9.44E-05 | 0.0766 | 140 | 149 | TAATAATAAT |
| Sme2.5_03738.1_g00001.1 | C2C2-YABBY | MP00620 | Sme2.5_13923.1_g00001.1 | CHS | 9.44E-05 | 0.0766 | 877 | 886 | TAATAATAAT |
| Sme2.5_03738.1_g00001.1 | C2C2-YABBY | MP00620 | Sme2.5_01401.1_g00004.1 | DFR | 4.98E-05 | 0.0691 | 1238 | 1247 | CAATAATAAC |
| Sme2.5_03738.1_g00001.1 | C2C2-YABBY | MP00620 | Sme2.5_01772.1_g00002.1 | F3'H | 6.11E-05 | 0.0766 | 1073 | 1082 | CAATTATAAT |
| Sme2.5_03738.1_g00001.1 | C2C2-YABBY | MP00620 | Sme2.5_16832.1_g00002.1 | PAL | 9.44E-05 | 0.0766 | 176 | 185 | TAATAATAAT |
| Sme2.5_03738.1_g00001.1 | C2C2-YABBY | MP00620 | Sme2.5_16832.1_g00002.1 | PAL | 9.44E-05 | 0.0766 | 179 | 188 | TAATAATAAT |
| Sme2.5_02180.1_g00004.1 | TCP | MP00065 | Sme2.5_11682.1_g00004.1 | PAL | 2.95E-06 | 0.0989 | 1358 | 1365 | GGGACCAC |
| Sme2.5_02180.1_g00004.1 | TCP | MP00065 | Sme2.5_01772.1_g00002.1 | F3'H | 8.48E-06 | 0.0989 | 1336 | 1343 | GGGACCAT |
| Sme2.5_02180.1_g00004.1 | TCP | MP00065 | Sme2.5_02148.1_g00009.1 | 5GT | 8.48E-06 | 0.0989 | 1374 | 1381 | GGGACCAT |
| Sme2.5_02180.1_g00004.1 | TCP | MP00065 | Sme2.5_01401.1_g00004.1 | DFR | 1.77E-05 | 0.143 | 543 | 550 | GGGACCAG |
| Sme2.5_02180.1_g00004.1 | TCP | MP00065 | Sme2.5_01638.1_g00005.1 | ANS | 3.21E-05 | 0.16 | 469 | 476 | GGGACCAA |
| Sme2.5_02180.1_g00004.1 | TCP | MP00065 | Sme2.5_01638.1_g00005.1 | ANS | 3.21E-05 | 0.16 | 473 | 480 | GGGACCAA |
| Sme2.5_02180.1_g00004.1 | TCP | MP00065 | Sme2.5_00188.1_g00020.1 | CHI | 4.39E-05 | 0.184 | 634 | 641 | GGGAGCAC |
| Sme2.5_02180.1_g00004.1 | TCP | MP00065 | Sme2.5_00188.1_g00020.1 | CHI | 4.39E-05 | 0.184 | 644 | 651 | GGGAGCAC |
| Sme2.5_02180.1_g00004.1 | TCP | MP00065 | Sme2.5_01193.1_g00009.1 | CHI | 9.92E-05 | 0.204 | 69 | 76 | GGGACTAC |
| Sme2.5_02180.1_g00004.1 | TCP | MP00065 | Sme2.5_01193.1_g00009.1 | CHI | 9.92E-05 | 0.204 | 81 | 88 | GGGACTAC |
| Sme2.5_02180.1_g00004.1 | TCP | MP00065 | Sme2.5_01193.1_g00009.1 | CHI | 9.92E-05 | 0.204 | 301 | 308 | GGGAACAC |
| Sme2.5_02180.1_g00004.1 | TCP | MP00065 | Sme2.5_00096.1_g00007.1 | 3GT | 9.92E-05 | 0.204 | 1237 | 1244 | GAGACCAC |
| Sme2.5_02180.1_g00004.1 | TCP | MP00065 | Sme2.5_06210.1_g00004.1 | 3GT | 9.92E-05 | 0.204 | 1400 | 1407 | GAGACCAC |
| Sme2.5_02180.1_g00004.1 | TCP | MP00065 | Sme2.5_04313.1_g00001.1 | F3'5'H | 9.92E-05 | 0.204 | 756 | 763 | GGGACTAC |
| Sme2.5_02180.1_g00004.1 | TCP | MP00065 | Sme2.5_01772.1_g00002.1 | F3'H | 9.92E-05 | 0.204 | 1523 | 1530 | GGGACAAC |
| Sme2.5_08041.1_g00002.1 | MYB4 | MP00479 | Sme2.5_00096.1_g00007.1 | 3GT | 3.34E-06 | 0.0239 | 134 | 148 | AAATTGGTAGGTGAA |
| Sme2.5_08041.1_g00002.1 | MYB4 | MP00479 | Sme2.5_00096.1_g00007.1 | 3GT | 4.74E-05 | 0.0677 | 901 | 915 | TTTCAAGTAGGTGGC |
| Sme2.5_08041.1_g00002.1 | MYB4 | MP00479 | Sme2.5_00228.1_g00013.1 | 3GT | 3.26E-05 | 0.0615 | 478 | 492 | TGATGGTTTGGTGGA |
| Sme2.5_08041.1_g00002.1 | MYB4 | MP00479 | Sme2.5_02148.1_g00009.1 | 5GT | 6.34E-05 | 0.0736 | 1343 | 1357 | AATATTTTTGGTGAG |
| Sme2.5_08041.1_g00002.1 | MYB4 | MP00479 | Sme2.5_01638.1_g00005.1 | ANS | 5.81E-05 | 0.0693 | 725 | 739 | ACACATGTTGGTGGA |
| Sme2.5_08041.1_g00002.1 | MYB4 | MP00479 | Sme2.5_01193.1_g00009.1 | CHI | 4.95E-05 | 0.0679 | 985 | 999 | TAGAGGTTTGGTGGT |
| Sme2.5_08041.1_g00002.1 | MYB4 | MP00479 | Sme2.5_01193.1_g00009.1 | CHI | 9.57E-05 | 0.0856 | 1330 | 1344 | CGGCAATTTGGTGGA |
| Sme2.5_08041.1_g00002.1 | MYB4 | MP00479 | Sme2.5_01193.1_g00009.1 | CHI | 9.57E-05 | 0.0856 | 1342 | 1356 | CGGCAATTTGGTGGA |
| Sme2.5_08041.1_g00002.1 | MYB4 | MP00479 | Sme2.5_13923.1_g00001.1 | CHS | 5.56E-05 | 0.0679 | 78 | 92 | ATAGTGTTAGGTGCA |
| Sme2.5_08041.1_g00002.1 | MYB4 | MP00479 | Sme2.5_13923.1_g00001.1 | CHS | 7.14E-05 | 0.0745 | 1365 | 1379 | GAGAAAGTAGGTAGC |
| Sme2.5_08041.1_g00002.1 | MYB4 | MP00479 | Sme2.5_13923.1_g00001.1 | CHS | 7.75E-05 | 0.0792 | 196 | 210 | ACTTAATTTGGTAAA |
| Sme2.5_08041.1_g00002.1 | MYB4 | MP00479 | Sme2.5_02154.1_g00001.1 | CHS | 4.44E-05 | 0.0677 | 486 | 500 | TAGTTGTTTGGTAAA |
| Sme2.5_08041.1_g00002.1 | MYB4 | MP00479 | Sme2.5_02154.1_g00001.1 | CHS | 8.16E-05 | 0.0809 | 1116 | 1130 | TGAGAAGTAGGTAGC |
| Sme2.5_08041.1_g00002.1 | MYB4 | MP00479 | Sme2.5_04313.1_g00001.1 | F3'5'H | 0.000051 | 0.0679 | 604 | 618 | CCTTAAGTAGGTAAC |
| Sme2.5_08041.1_g00002.1 | MYB4 | MP00479 | Sme2.5_04313.1_g00001.1 | F3'5'H | 6.95E-05 | 0.074 | 1402 | 1416 | TAGGGATTTGGTAGG |
| Sme2.5_08041.1_g00002.1 | MYB4 | MP00479 | Sme2.5_00015.1_g00020.1 | F3H | 7.07E-06 | 0.0354 | 827 | 841 | GTAGAGTTAGGTGGA |
| Sme2.5_08041.1_g00002.1 | MYB4 | MP00479 | Sme2.5_01772.1_g00002.1 | F3'H | 9.47E-06 | 0.0395 | 698 | 712 | CGTTTGGTTGGTAAG |
| Sme2.5_08041.1_g00002.1 | MYB4 | MP00479 | Sme2.5_01772.1_g00002.1 | F3'H | 0.000043 | 0.0677 | 641 | 655 | TAATTATTTGGTGAG |
| Sme2.5_08041.1_g00002.1 | MYB4 | MP00479 | Sme2.5_04927.1_g00001.1 | F3'H | 3.26E-05 | 0.0615 | 513 | 527 | ATAATTGTTGGTGAG |
| Sme2.5_08041.1_g00002.1 | MYB4 | MP00479 | Sme2.5_04927.1_g00001.1 | F3'H | 6.77E-05 | 0.0736 | 675 | 689 | GTAAGGTTAGGTGGT |
| Sme2.5_08041.1_g00002.1 | MYB4 | MP00479 | Sme2.5_16832.1_g00002.1 | PAL | 3.84E-06 | 0.024 | 1178 | 1192 | TTCGGGGTTGGTGGA |
| Sme2.5_08041.1_g00002.1 | MYB4 | MP00479 | Sme2.5_00209.1_g00001.1 | PAL | 4.8E-06 | 0.0267 | 1283 | 1297 | TTGGGGGTTGGTGGA |
| Sme2.5_08041.1_g00002.1 | MYB4 | MP00479 | Sme2.5_16832.1_g00002.1 | PAL | 1.46E-05 | 0.0487 | 1353 | 1367 | ATGATGGTAGGTAAA |
| Sme2.5_08041.1_g00002.1 | MYB4 | MP00479 | Sme2.5_00209.1_g00001.1 | PAL | 2.56E-05 | 0.0615 | 398 | 412 | TTGTTGTTTGGTAGA |
| Sme2.5_08041.1_g00002.1 | MYB4 | MP00479 | Sme2.5_00209.1_g00001.1 | PAL | 3.04E-05 | 0.0615 | 986 | 1000 | GTAGAGTTAGGTGGT |
| Sme2.5_08041.1_g00002.1 | MYB4 | MP00479 | Sme2.5_00209.1_g00001.1 | PAL | 4.66E-05 | 0.0677 | 996 | 1010 | TAATATGTTGGTAGA |
| Sme2.5_08041.1_g00002.1 | MYB4 | MP00479 | Sme2.5_16832.1_g00002.1 | PAL | 5.48E-05 | 0.0679 | 59 | 73 | ACTCAATTTGGTGGA |
| Sme2.5_08041.1_g00002.1 | MYB4 | MP00479 | Sme2.5_00209.1_g00001.1 | PAL | 8.36E-05 | 0.0809 | 1444 | 1458 | GGGATGGTAGGTAAA |
| Sme2.5_08041.1_g00002.1 | MYB4 | MP00479 | Sme2.5_00864.1_g00009.1 | PAL | 1.18E-05 | 0.0456 | 1152 | 1166 | CAATGAGTTGGTGAG |
| Sme2.5_08041.1_g00002.1 | MYB4 | MP00479 | Sme2.5_00864.1_g00009.1 | PAL | 4.44E-05 | 0.0677 | 272 | 286 | ATTTAGTTTGGTAAT |
| Sme2.5_08041.1_g00002.1 | MYB4 | MP00479 | Sme2.5_00864.1_g00009.1 | PAL | 5.56E-05 | 0.0679 | 906 | 920 | GAGTAATTTGGTAGG |
| Sme2.5_08041.1_g00002.1 | MYB4 | MP00479 | Sme2.5_11682.1_g00004.1 | PAL | 8.88E-06 | 0.0395 | 116 | 130 | CTTTGGTTTGGTGGC |
| Sme2.5_08041.1_g00002.1 | MYB4 | MP00479 | Sme2.5_11682.1_g00004.1 | PAL | 8.99E-05 | 0.0834 | 289 | 303 | CATCAGTTTGGTATG |
| Sme2.5_02806.1_g00001.1 | zf-HD | MP00387 | Sme2.5_00228.1_g00013.1 | 3GT | 6.08E-06 | 0.0237 | 1389 | 1403 | TTTATTAATTAATTA |
| Sme2.5_02806.1_g00001.1 | zf-HD | MP00387 | Sme2.5_00228.1_g00013.1 | 3GT | 2.09E-05 | 0.0518 | 1398 | 1412 | ATTAAATCCTAATTA |
| Sme2.5_02806.1_g00001.1 | zf-HD | MP00387 | Sme2.5_00228.1_g00013.1 | 3GT | 4.14E-05 | 0.0715 | 330 | 344 | TATATTAGTTAATTA |
| Sme2.5_02806.1_g00001.1 | zf-HD | MP00387 | Sme2.5_00228.1_g00013.1 | 3GT | 5.37E-05 | 0.0732 | 1403 | 1417 | ACTAGTCATTAATCA |
| Sme2.5_02806.1_g00001.1 | zf-HD | MP00387 | Sme2.5_00228.1_g00013.1 | 3GT | 6.47E-05 | 0.0784 | 618 | 632 | ATAAAAAATTAATTA |
| Sme2.5_02806.1_g00001.1 | zf-HD | MP00387 | Sme2.5_00843.1_g00005.1 | 4CL | 6.08E-06 | 0.0237 | 189 | 203 | ATTCTTCACTAATCA |
| Sme2.5_02806.1_g00001.1 | zf-HD | MP00387 | Sme2.5_02148.1_g00009.1 | 5GT | 2.98E-06 | 0.0206 | 1042 | 1056 | TTTAGTACTTAATTA |
| Sme2.5_02806.1_g00001.1 | zf-HD | MP00387 | Sme2.5_01638.1_g00005.1 | ANS | 2.02E-05 | 0.0518 | 349 | 363 | ATTATCTATTAATTA |
| Sme2.5_02806.1_g00001.1 | zf-HD | MP00387 | Sme2.5_00188.1_g00020.1 | CHI | 5.29E-05 | 0.0732 | 1269 | 1283 | ATAGGCCGTTAATTA |
| Sme2.5_02806.1_g00001.1 | zf-HD | MP00387 | Sme2.5_00188.1_g00020.1 | CHI | 5.29E-05 | 0.0732 | 1279 | 1293 | ATAGGCCGTTAATTA |
| Sme2.5_02806.1_g00001.1 | zf-HD | MP00387 | Sme2.5_01193.1_g00009.1 | CHI | 9.25E-05 | 0.0852 | 901 | 915 | TTTGAGACTTAATTA |
| Sme2.5_02806.1_g00001.1 | zf-HD | MP00387 | Sme2.5_01193.1_g00009.1 | CHI | 9.25E-05 | 0.0852 | 913 | 927 | TTTGAGACTTAATTA |
| Sme2.5_02806.1_g00001.1 | zf-HD | MP00387 | Sme2.5_01193.1_g00009.1 | CHI | 9.85E-05 | 0.0852 | 1233 | 1247 | AAAATACATTAATTA |
| Sme2.5_02806.1_g00001.1 | zf-HD | MP00387 | Sme2.5_01193.1_g00009.1 | CHI | 9.85E-05 | 0.0852 | 1245 | 1259 | AAAATACATTAATTA |
| Sme2.5_02806.1_g00001.1 | zf-HD | MP00387 | Sme2.5_13923.1_g00001.1 | CHS | 8.21E-05 | 0.0843 | 1333 | 1347 | ATTTTTGGTTAATTA |
| Sme2.5_02806.1_g00001.1 | zf-HD | MP00387 | Sme2.5_02154.1_g00001.1 | CHS | 5.53E-05 | 0.0734 | 887 | 901 | TTTTGACATTAATTA |
| Sme2.5_02806.1_g00001.1 | zf-HD | MP00387 | Sme2.5_02154.1_g00001.1 | CHS | 6.37E-05 | 0.0784 | 547 | 561 | AAAAATTCTTAATCA |
| Sme2.5_02806.1_g00001.1 | zf-HD | MP00387 | Sme2.5_04313.1_g00001.1 | F3'5'H | 1.25E-05 | 0.036 | 227 | 241 | TTTCTTTCCTAATTA |
| Sme2.5_02806.1_g00001.1 | zf-HD | MP00387 | Sme2.5_04313.1_g00001.1 | F3'5'H | 7.87E-05 | 0.0843 | 218 | 232 | AATATTTATTAATTA |
| Sme2.5_02806.1_g00001.1 | zf-HD | MP00387 | Sme2.5_01772.1_g00002.1 | F3'H | 4.59E-07 | 0.00512 | 651 | 665 | TTTATTCGTTAATCA |
| Sme2.5_02806.1_g00001.1 | zf-HD | MP00387 | Sme2.5_01772.1_g00002.1 | F3'H | 1.38E-05 | 0.0377 | 1210 | 1224 | TTTTATCATTAATTA |
| Sme2.5_02806.1_g00001.1 | zf-HD | MP00387 | Sme2.5_01772.1_g00002.1 | F3'H | 8.12E-05 | 0.0843 | 20 | 34 | ATAATGAACTAATTA |
| Sme2.5_02806.1_g00001.1 | zf-HD | MP00387 | Sme2.5_01772.1_g00002.1 | F3'H | 9.85E-05 | 0.0852 | 1107 | 1121 | AAAATTAATTAATTA |
| Sme2.5_02806.1_g00001.1 | zf-HD | MP00387 | Sme2.5_04927.1_g00001.1 | F3'H | 9.54E-05 | 0.0852 | 1008 | 1022 | TAAACTTGCTAATTA |
| Sme2.5_02806.1_g00001.1 | zf-HD | MP00387 | Sme2.5_00209.1_g00001.1 | PAL | 4.73E-05 | 0.0715 | 83 | 97 | TTTGTCGATTAATTA |
| Sme2.5_00537.1_g00003.1 | BIM1 | MP00498 | Sme2.5_00096.1_g00007.1 | 3GT | 7.09E-05 | 0.0737 | 627 | 640 | CACGTGTGGGTTGT |
| Sme2.5_00537.1_g00003.1 | BIM1 | MP00498 | Sme2.5_00843.1_g00005.1 | 4CL | 3.23E-05 | 0.0634 | 1524 | 1537 | CACGTGAATCCGCC |
| Sme2.5_00537.1_g00003.1 | BIM1 | MP00498 | Sme2.5_00843.1_g00005.1 | 4CL | 5.48E-05 | 0.071 | 1516 | 1529 | CACGTGGTCTGTGT |
| Sme2.5_00537.1_g00003.1 | BIM1 | MP00498 | Sme2.5_02148.1_g00009.1 | 5GT | 1.48E-06 | 0.0148 | 87 | 100 | CACGTGACAATCAA |
| Sme2.5_00537.1_g00003.1 | BIM1 | MP00498 | Sme2.5_01638.1_g00005.1 | ANS | 8.88E-08 | 0.00101 | 434 | 447 | CACGTGACAAGCAC |
| Sme2.5_00537.1_g00003.1 | BIM1 | MP00498 | Sme2.5_01638.1_g00005.1 | ANS | 8.88E-08 | 0.00101 | 438 | 451 | CACGTGACAAGCAC |
| Sme2.5_00537.1_g00003.1 | BIM1 | MP00498 | Sme2.5_01638.1_g00005.1 | ANS | 3.79E-06 | 0.0202 | 1392 | 1405 | CACGTGACTCATAG |
| Sme2.5_00537.1_g00003.1 | BIM1 | MP00498 | Sme2.5_01638.1_g00005.1 | ANS | 3.79E-06 | 0.0202 | 1392 | 1405 | CACGTGACTCATAG |
| Sme2.5_00537.1_g00003.1 | BIM1 | MP00498 | Sme2.5_01638.1_g00005.1 | ANS | 3.79E-06 | 0.0202 | 1396 | 1409 | CACGTGACTCATAG |
| Sme2.5_00537.1_g00003.1 | BIM1 | MP00498 | Sme2.5_01638.1_g00005.1 | ANS | 4.47E-05 | 0.0634 | 76 | 89 | CACGTGGTGTTCAA |
| Sme2.5_00537.1_g00003.1 | BIM1 | MP00498 | Sme2.5_01638.1_g00005.1 | ANS | 4.47E-05 | 0.0634 | 80 | 93 | CACGTGGTGTTCAA |
| Sme2.5_00537.1_g00003.1 | BIM1 | MP00498 | Sme2.5_01638.1_g00005.1 | ANS | 6.86E-05 | 0.0722 | 442 | 455 | CACGTGGTTGTTCA |
| Sme2.5_00537.1_g00003.1 | BIM1 | MP00498 | Sme2.5_01638.1_g00005.1 | ANS | 6.86E-05 | 0.0722 | 446 | 459 | CACGTGGTTGTTCA |
| Sme2.5_00537.1_g00003.1 | BIM1 | MP00498 | Sme2.5_01638.1_g00005.1 | ANS | 8.93E-05 | 0.0784 | 1086 | 1099 | CACATGAGGTTCAT |
| Sme2.5_00537.1_g00003.1 | BIM1 | MP00498 | Sme2.5_13923.1_g00001.1 | CHS | 2.64E-06 | 0.0202 | 1410 | 1423 | CACGTGCCACTAGC |
| Sme2.5_00537.1_g00003.1 | BIM1 | MP00498 | Sme2.5_13923.1_g00001.1 | CHS | 4.88E-06 | 0.0243 | 1402 | 1415 | CACGTGACCCTTTT |
| Sme2.5_00537.1_g00003.1 | BIM1 | MP00498 | Sme2.5_13923.1_g00001.1 | CHS | 8.12E-06 | 0.0243 | 1432 | 1445 | CACGTGATCTCTAA |
| Sme2.5_00537.1_g00003.1 | BIM1 | MP00498 | Sme2.5_13923.1_g00001.1 | CHS | 8.96E-06 | 0.0247 | 1424 | 1437 | CACGTGCCTCAACA |
| Sme2.5_00537.1_g00003.1 | BIM1 | MP00498 | Sme2.5_02154.1_g00001.1 | CHS | 6.54E-05 | 0.0722 | 1427 | 1440 | CACGTGAATACTAA |
| Sme2.5_00537.1_g00003.1 | BIM1 | MP00498 | Sme2.5_02154.1_g00001.1 | CHS | 7.57E-05 | 0.0756 | 1419 | 1432 | CACGTGGTAGAGCA |
| Sme2.5_00537.1_g00003.1 | BIM1 | MP00498 | Sme2.5_01401.1_g00004.1 | DFR | 6.81E-06 | 0.0243 | 1411 | 1424 | CACGTGCTCAACGC |
| Sme2.5_00537.1_g00003.1 | BIM1 | MP00498 | Sme2.5_01401.1_g00004.1 | DFR | 3.79E-05 | 0.0634 | 1419 | 1432 | CACGTGGTTACCAT |
| Sme2.5_00537.1_g00003.1 | BIM1 | MP00498 | Sme2.5_00015.1_g00020.1 | F3H | 2.36E-05 | 0.0589 | 549 | 562 | CACGTGAGGTATAA |
| Sme2.5_00537.1_g00003.1 | BIM1 | MP00498 | Sme2.5_00015.1_g00020.1 | F3H | 3.12E-05 | 0.0634 | 1385 | 1398 | CACGTGCTTCTTTT |
| Sme2.5_00537.1_g00003.1 | BIM1 | MP00498 | Sme2.5_00015.1_g00020.1 | F3H | 6.13E-05 | 0.071 | 541 | 554 | CACGTGTGAGATTC |
| Sme2.5_00669.1_g00015.1 | MYB94 | MP00395 | Sme2.5_00096.1_g00007.1 | 3GT | 1.57E-05 | 0.024 | 156 | 174 | TTTTGGGTGGTTGAAAGTT |
| Sme2.5_00669.1_g00015.1 | MYB94 | MP00395 | Sme2.5_00096.1_g00007.1 | 3GT | 5.83E-05 | 0.0572 | 130 | 148 | AAATTGGTAGGTGAATAGA |
| Sme2.5_00669.1_g00015.1 | MYB94 | MP00395 | Sme2.5_00096.1_g00007.1 | 3GT | 6.02E-05 | 0.0572 | 1215 | 1233 | GGTATATTAGTTGAGCAGG |
| Sme2.5_00669.1_g00015.1 | MYB94 | MP00395 | Sme2.5_00228.1_g00013.1 | 3GT | 9.29E-06 | 0.0226 | 722 | 740 | AGGATGGTGGGTGTTAAGA |
| Sme2.5_00669.1_g00015.1 | MYB94 | MP00395 | Sme2.5_00228.1_g00013.1 | 3GT | 9.82E-06 | 0.0226 | 408 | 426 | GGACTAGTGGTTGGCCCAT |
| Sme2.5_00669.1_g00015.1 | MYB94 | MP00395 | Sme2.5_00843.1_g00005.1 | 4CL | 6.17E-05 | 0.0572 | 32 | 50 | AAAAAGTTGGTTGTAAAGA |
| Sme2.5_00669.1_g00015.1 | MYB94 | MP00395 | Sme2.5_02148.1_g00009.1 | 5GT | 7.41E-06 | 0.0226 | 1440 | 1458 | TTTTGGGTGGGTGGTGGAG |
| Sme2.5_00669.1_g00015.1 | MYB94 | MP00395 | Sme2.5_02148.1_g00009.1 | 5GT | 7.51E-05 | 0.0632 | 1430 | 1448 | GTGGTGGAGGGTGGCACAT |
| Sme2.5_00669.1_g00015.1 | MYB94 | MP00395 | Sme2.5_02148.1_g00009.1 | 5GT | 8.55E-05 | 0.067 | 1436 | 1454 | GGGTGGGTGGTGGAGGGTG |
| Sme2.5_00669.1_g00015.1 | MYB94 | MP00395 | Sme2.5_02148.1_g00009.1 | 5GT | 0.000097 | 0.0713 | 1243 | 1261 | AACGGGTTGGGTGGAATTG |
| Sme2.5_00669.1_g00015.1 | MYB94 | MP00395 | Sme2.5_01638.1_g00005.1 | ANS | 1.23E-05 | 0.0226 | 1408 | 1426 | TGCATGGTGGTTGAATGTT |
| Sme2.5_00669.1_g00015.1 | MYB94 | MP00395 | Sme2.5_01638.1_g00005.1 | ANS | 1.23E-05 | 0.0226 | 1412 | 1430 | TGCATGGTGGTTGAATGTT |
| Sme2.5_00669.1_g00015.1 | MYB94 | MP00395 | Sme2.5_01638.1_g00005.1 | ANS | 1.38E-05 | 0.0226 | 1314 | 1332 | ATAATGGTGGTTGGCTCAC |
| Sme2.5_00669.1_g00015.1 | MYB94 | MP00395 | Sme2.5_01638.1_g00005.1 | ANS | 1.38E-05 | 0.0226 | 1318 | 1336 | ATAATGGTGGTTGGCTCAC |
| Sme2.5_00669.1_g00015.1 | MYB94 | MP00395 | Sme2.5_07446.1_g00004.1 | ATP | 5.51E-05 | 0.0572 | 500 | 518 | TTAGTATTAGTTGGAAATT |
| Sme2.5_00669.1_g00015.1 | MYB94 | MP00395 | Sme2.5_01193.1_g00009.1 | CHI | 2.12E-06 | 0.0226 | 989 | 1007 | GGTTTGGTGGTTGAAGGAA |
| Sme2.5_00669.1_g00015.1 | MYB94 | MP00395 | Sme2.5_01193.1_g00009.1 | CHI | 6.37E-05 | 0.0572 | 1307 | 1325 | GGAAAGTTGGGTGGTTTAA |
| Sme2.5_00669.1_g00015.1 | MYB94 | MP00395 | Sme2.5_01193.1_g00009.1 | CHI | 6.37E-05 | 0.0572 | 1319 | 1337 | GGAAAGTTGGGTGGTTTAA |
| Sme2.5_00669.1_g00015.1 | MYB94 | MP00395 | Sme2.5_13923.1_g00001.1 | CHS | 6.17E-05 | 0.0572 | 1365 | 1383 | GAGAAAGTAGGTAGCAGTC |
| Sme2.5_00669.1_g00015.1 | MYB94 | MP00395 | Sme2.5_01401.1_g00004.1 | DFR | 2.26E-05 | 0.0302 | 1359 | 1377 | TGACTGGTTGTTGAGAGGA |
| Sme2.5_00669.1_g00015.1 | MYB94 | MP00395 | Sme2.5_01401.1_g00004.1 | DFR | 5.24E-05 | 0.0572 | 886 | 904 | ACTACAGTAGTTGGCACTT |
| Sme2.5_00669.1_g00015.1 | MYB94 | MP00395 | Sme2.5_04313.1_g00001.1 | F3'5'H | 0.000015 | 0.0238 | 928 | 946 | GTTGAAGTAGTTGAAAAAA |
| Sme2.5_00669.1_g00015.1 | MYB94 | MP00395 | Sme2.5_00015.1_g00020.1 | F3H | 3.18E-05 | 0.0415 | 1418 | 1436 | CGAGTGGTGGTTAGAGCTC |
| Sme2.5_00669.1_g00015.1 | MYB94 | MP00395 | Sme2.5_00015.1_g00020.1 | F3H | 8.61E-05 | 0.067 | 224 | 242 | CTAATATTGGTTGGCACTA |
| Sme2.5_00669.1_g00015.1 | MYB94 | MP00395 | Sme2.5_00015.1_g00020.1 | F3H | 8.87E-05 | 0.0671 | 823 | 841 | GTAGAGTTAGGTGGATATC |
| Sme2.5_00669.1_g00015.1 | MYB94 | MP00395 | Sme2.5_01638.1_g00003.1 | LDOX | 1.98E-06 | 0.0226 | 487 | 505 | GCGTTGTTGGTTGGAGGGT |
| Sme2.5_00669.1_g00015.1 | MYB94 | MP00395 | Sme2.5_01638.1_g00003.1 | LDOX | 8.87E-05 | 0.0671 | 515 | 533 | GGGGAGGTGGTGGAGCTAT |
| Sme2.5_00669.1_g00015.1 | MYB94 | MP00395 | Sme2.5_16832.1_g00002.1 | PAL | 5.44E-06 | 0.0226 | 1299 | 1317 | ATGGTGGTTGTTGGAAGAT |
| Sme2.5_00669.1_g00015.1 | MYB94 | MP00395 | Sme2.5_16832.1_g00002.1 | PAL | 9.93E-06 | 0.0226 | 1302 | 1320 | AAAATGGTGGTTGTTGGAA |
| Sme2.5_00669.1_g00015.1 | MYB94 | MP00395 | Sme2.5_16832.1_g00002.1 | PAL | 1.29E-05 | 0.0226 | 1147 | 1165 | TTTTTGGTTGTTGGGAGAA |
| Sme2.5_00669.1_g00015.1 | MYB94 | MP00395 | Sme2.5_00209.1_g00001.1 | PAL | 3.83E-05 | 0.0488 | 1252 | 1270 | TTTGGAGTTGTTGGGAGAA |
| Sme2.5_00669.1_g00015.1 | MYB94 | MP00395 | Sme2.5_00209.1_g00001.1 | PAL | 4.67E-05 | 0.0566 | 982 | 1000 | GTAGAGTTAGGTGGTGTCT |
| Sme2.5_00669.1_g00015.1 | MYB94 | MP00395 | Sme2.5_16832.1_g00002.1 | PAL | 6.12E-05 | 0.0572 | 333 | 351 | GGGGGGGTGGGGGGGCGGA |
| Sme2.5_00669.1_g00015.1 | MYB94 | MP00395 | Sme2.5_00209.1_g00001.1 | PAL | 6.27E-05 | 0.0572 | 1440 | 1458 | GGGATGGTAGGTAAAGAAA |
| Sme2.5_00669.1_g00015.1 | MYB94 | MP00395 | Sme2.5_11682.1_g00004.1 | PAL | 4.29E-05 | 0.0533 | 853 | 871 | GATGTGTTTGTTGGGCGGT |
| Sme2.5_00669.1_g00015.1 | MYB94 | MP00395 | Sme2.5_11682.1_g00004.1 | PAL | 5.55E-05 | 0.0572 | 36 | 54 | TCAAAGTTGGTTGGATACG |
| Sme2.5_00669.1_g00015.1 | MYB94 | MP00395 | Sme2.5_11682.1_g00004.1 | PAL | 0.00007 | 0.0599 | 956 | 974 | ATGCAGGTGGGTAGTCAGT |
| Sme2.5_00962.1_g00008.1 | MYB15 | MP00375 | Sme2.5_00096.1_g00007.1 | 3GT | 2.85E-05 | 0.0404 | 124 | 144 | GAAAGGTCTATTCACCTACCA |
| Sme2.5_00962.1_g00008.1 | MYB15 | MP00375 | Sme2.5_00096.1_g00007.1 | 3GT | 8.26E-05 | 0.0583 | 151 | 171 | CAAAAAACTTTCAACCACCCA |
| Sme2.5_00962.1_g00008.1 | MYB15 | MP00375 | Sme2.5_00096.1_g00007.1 | 3GT | 8.81E-05 | 0.0592 | 919 | 939 | TCCAAACGCAATCACCAAATT |
| Sme2.5_00962.1_g00008.1 | MYB15 | MP00375 | Sme2.5_00096.1_g00007.1 | 3GT | 8.97E-05 | 0.0592 | 891 | 911 | GTTGGCATAAGCCACCTACTT |
| Sme2.5_00962.1_g00008.1 | MYB15 | MP00375 | Sme2.5_06210.1_g00004.1 | 3GT | 8.97E-05 | 0.0592 | 1273 | 1293 | ACCCCATAGCTTCACCACATA |
| Sme2.5_00962.1_g00008.1 | MYB15 | MP00375 | Sme2.5_00228.1_g00013.1 | 3GT | 9.33E-06 | 0.0344 | 720 | 740 | CTTCTTAACACCCACCATCCT |
| Sme2.5_00962.1_g00008.1 | MYB15 | MP00375 | Sme2.5_00228.1_g00013.1 | 3GT | 2.88E-05 | 0.0404 | 482 | 502 | ATAGATTAAATCCACCAAACC |
| Sme2.5_00962.1_g00008.1 | MYB15 | MP00375 | Sme2.5_02148.1_g00009.1 | 5GT | 6.96E-06 | 0.0337 | 1431 | 1451 | TGTGCCACCCTCCACCACCCA |
| Sme2.5_00962.1_g00008.1 | MYB15 | MP00375 | Sme2.5_02148.1_g00009.1 | 5GT | 1.69E-05 | 0.0344 | 1246 | 1266 | CCGCCCAATTCCACCCAACCC |
| Sme2.5_00962.1_g00008.1 | MYB15 | MP00375 | Sme2.5_02148.1_g00009.1 | 5GT | 8.81E-05 | 0.0592 | 1347 | 1367 | ATTGCTCAACCTCACCAAAAA |
| Sme2.5_00962.1_g00008.1 | MYB15 | MP00375 | Sme2.5_01638.1_g00005.1 | ANS | 1.46E-05 | 0.0344 | 729 | 749 | CCACTCGTTTTCCACCAACAT |
| Sme2.5_00962.1_g00008.1 | MYB15 | MP00375 | Sme2.5_01638.1_g00005.1 | ANS | 0.000055 | 0.0477 | 1053 | 1073 | CAAGACCAGCACCACCAATCT |
| Sme2.5_00962.1_g00008.1 | MYB15 | MP00375 | Sme2.5_01638.1_g00005.1 | ANS | 9.64E-05 | 0.0604 | 1530 | 1550 | ATATAGACTGAACACCAACCC |
| Sme2.5_00962.1_g00008.1 | MYB15 | MP00375 | Sme2.5_01193.1_g00009.1 | CHI | 1.01E-05 | 0.0344 | 989 | 1009 | TTTTCCTTCAACCACCAAACC |
| Sme2.5_00962.1_g00008.1 | MYB15 | MP00375 | Sme2.5_01193.1_g00009.1 | CHI | 4.21E-05 | 0.0441 | 1334 | 1354 | TGTCAGTCACTCCACCAAATT |
| Sme2.5_00962.1_g00008.1 | MYB15 | MP00375 | Sme2.5_01193.1_g00009.1 | CHI | 4.21E-05 | 0.0441 | 1346 | 1366 | TGTCAGTCACTCCACCAAATT |
| Sme2.5_00962.1_g00008.1 | MYB15 | MP00375 | Sme2.5_02154.1_g00001.1 | CHS | 4.38E-05 | 0.0447 | 1120 | 1140 | CGCCACTAATGCTACCTACTT |
| Sme2.5_00962.1_g00008.1 | MYB15 | MP00375 | Sme2.5_01401.1_g00004.1 | DFR | 0.000047 | 0.0447 | 1353 | 1373 | TTTTCTTCCTCTCAACAACCA |
| Sme2.5_00962.1_g00008.1 | MYB15 | MP00375 | Sme2.5_04313.1_g00001.1 | F3'5'H | 0.00003 | 0.0404 | 327 | 347 | TCCCCCCCCCCCCCCCAAATA |
| Sme2.5_00962.1_g00008.1 | MYB15 | MP00375 | Sme2.5_04313.1_g00001.1 | F3'5'H | 4.75E-05 | 0.0447 | 432 | 452 | AGGTCAGACATCAACCTACCC |
| Sme2.5_00962.1_g00008.1 | MYB15 | MP00375 | Sme2.5_04313.1_g00001.1 | F3'5'H | 5.66E-05 | 0.0484 | 1302 | 1322 | CTAAGGCTTTTCCACCTAGCA |
| Sme2.5_00962.1_g00008.1 | MYB15 | MP00375 | Sme2.5_00015.1_g00020.1 | F3H | 4.09E-05 | 0.0441 | 817 | 837 | AGGCCAGATATCCACCTAACT |
| Sme2.5_00962.1_g00008.1 | MYB15 | MP00375 | Sme2.5_01772.1_g00002.1 | F3'H | 2.37E-05 | 0.037 | 692 | 712 | TTTCCACTTACCAACCAAACG |
| Sme2.5_00962.1_g00008.1 | MYB15 | MP00375 | Sme2.5_01772.1_g00002.1 | F3'H | 0.000066 | 0.052 | 688 | 708 | ATATTTTCCACTTACCAACCA |
| Sme2.5_00962.1_g00008.1 | MYB15 | MP00375 | Sme2.5_01772.1_g00002.1 | F3'H | 9.64E-05 | 0.0604 | 645 | 665 | TACTACTTAGCTCACCAAATA |
| Sme2.5_00962.1_g00008.1 | MYB15 | MP00375 | Sme2.5_04927.1_g00001.1 | F3'H | 2.97E-05 | 0.0404 | 517 | 537 | ATGAAAAAATCTCACCAACAA |
| Sme2.5_00962.1_g00008.1 | MYB15 | MP00375 | Sme2.5_01638.1_g00003.1 | LDOX | 8.37E-06 | 0.0344 | 518 | 538 | CATCTATAGCTCCACCACCTC |
| Sme2.5_00962.1_g00008.1 | MYB15 | MP00375 | Sme2.5_01638.1_g00003.1 | LDOX | 9.79E-06 | 0.0344 | 515 | 535 | CTATAGCTCCACCACCTCCCC |
| Sme2.5_00962.1_g00008.1 | MYB15 | MP00375 | Sme2.5_01638.1_g00003.1 | LDOX | 1.91E-05 | 0.0344 | 490 | 510 | GGACAACCCTCCAACCAACAA |
| Sme2.5_00962.1_g00008.1 | MYB15 | MP00375 | Sme2.5_01638.1_g00003.1 | LDOX | 6.06E-05 | 0.0497 | 494 | 514 | CATCGGACAACCCTCCAACCA |
| Sme2.5_00962.1_g00008.1 | MYB15 | MP00375 | Sme2.5_16832.1_g00002.1 | PAL | 3.48E-07 | 0.0114 | 1168 | 1188 | AACTCCTAATTCCACCAACCC |
| Sme2.5_00962.1_g00008.1 | MYB15 | MP00375 | Sme2.5_00209.1_g00001.1 | PAL | 5.64E-07 | 0.0114 | 1273 | 1293 | CTATATTAAATCCACCAACCC |
| Sme2.5_00962.1_g00008.1 | MYB15 | MP00375 | Sme2.5_16832.1_g00002.1 | PAL | 2.33E-06 | 0.0325 | 327 | 347 | CCCCCCCACCCCCCCCACCCA |
| Sme2.5_00962.1_g00008.1 | MYB15 | MP00375 | Sme2.5_16832.1_g00002.1 | PAL | 1.18E-05 | 0.0344 | 1343 | 1363 | CTCTTATTTCTTTACCTACCA |
| Sme2.5_00962.1_g00008.1 | MYB15 | MP00375 | Sme2.5_16832.1_g00002.1 | PAL | 1.27E-05 | 0.0344 | 336 | 356 | CACCCTCCGCCCCCCCACCCC |
| Sme2.5_00962.1_g00008.1 | MYB15 | MP00375 | Sme2.5_16832.1_g00002.1 | PAL | 1.93E-05 | 0.0344 | 1141 | 1161 | TCACTTTTCTCCCAACAACCA |
| Sme2.5_00962.1_g00008.1 | MYB15 | MP00375 | Sme2.5_16832.1_g00002.1 | PAL | 1.95E-05 | 0.0344 | 1389 | 1409 | CACCACAAATCCCATCTACCA |
| Sme2.5_00962.1_g00008.1 | MYB15 | MP00375 | Sme2.5_00209.1_g00001.1 | PAL | 5.94E-05 | 0.0497 | 1480 | 1500 | CACCACATATCCCATCAAACA |
| Sme2.5_00962.1_g00008.1 | MYB15 | MP00375 | Sme2.5_00209.1_g00001.1 | PAL | 6.92E-05 | 0.0539 | 986 | 1006 | ACCACCTAACTCTACCAACAT |
| Sme2.5_00962.1_g00008.1 | MYB15 | MP00375 | Sme2.5_16832.1_g00002.1 | PAL | 7.05E-05 | 0.0542 | 1293 | 1313 | TAATTAATCTTCCAACAACCA |
| Sme2.5_00962.1_g00008.1 | MYB15 | MP00375 | Sme2.5_16832.1_g00002.1 | PAL | 7.74E-05 | 0.057 | 323 | 343 | CCCACCCCCCCCACCCAAACT |
| Sme2.5_00962.1_g00008.1 | MYB15 | MP00375 | Sme2.5_16832.1_g00002.1 | PAL | 7.81E-05 | 0.057 | 93 | 113 | CCTCGACCATCTCACCACAAC |
| Sme2.5_00962.1_g00008.1 | MYB15 | MP00375 | Sme2.5_00209.1_g00001.1 | PAL | 8.11E-05 | 0.0579 | 1434 | 1454 | ACATATTTTCTTTACCTACCA |
| Sme2.5_00962.1_g00008.1 | MYB15 | MP00375 | Sme2.5_00209.1_g00001.1 | PAL | 0.0001 | 0.0613 | 976 | 996 | AAGGCCAGACACCACCTAACT |
| Sme2.5_00962.1_g00008.1 | MYB15 | MP00375 | Sme2.5_16832.1_g00002.1 | PAL | 0.0001 | 0.0613 | 1399 | 1419 | CCCATCTACCACAACCAAAAA |
| Sme2.5_00962.1_g00008.1 | MYB15 | MP00375 | Sme2.5_00864.1_g00009.1 | PAL | 3.77E-05 | 0.0416 | 1142 | 1162 | ATAAATTTGACTCACCAACTC |
| Sme2.5_00962.1_g00008.1 | MYB15 | MP00375 | Sme2.5_11682.1_g00004.1 | PAL | 2.23E-05 | 0.0356 | 120 | 140 | ATGGCATAAAGCCACCAAACC |
| Sme2.5_00962.1_g00008.1 | MYB15 | MP00375 | Sme2.5_11682.1_g00004.1 | PAL | 3.36E-05 | 0.0416 | 39 | 59 | CCCGCCGTATCCAACCAACTT |
| Sme2.5_00622.1_g00022.1 | SPL9 | MP00307 | Sme2.5_00228.1_g00013.1 | 3GT | 0.000097 | 0.32 | 383 | 393 | TGTGTACGGAG |
| Sme2.5_00622.1_g00022.1 | SPL9 | MP00307 | Sme2.5_02154.1_g00001.1 | CHS | 7.36E-05 | 0.314 | 1159 | 1169 | ATTGTACTTAC |
| Sme2.5_00622.1_g00022.1 | SPL9 | MP00307 | Sme2.5_01401.1_g00004.1 | DFR | 1.01E-05 | 0.113 | 1458 | 1468 | CTTGTACGTAC |
| Sme2.5_00622.1_g00022.1 | SPL9 | MP00307 | Sme2.5_01401.1_g00004.1 | DFR | 3.23E-05 | 0.165 | 1455 | 1465 | CATGTACGTAC |
| Sme2.5_00622.1_g00022.1 | SPL9 | MP00307 | Sme2.5_04313.1_g00001.1 | F3'5'H | 1.73E-05 | 0.147 | 39 | 49 | ATTGTACTGAT |
| Sme2.5_02766.1_g00005.1 | AP2-EREBP | MP00302 | Sme2.5_06210.1_g00004.1 | 3GT | 1.61E-05 | 0.0652 | 1131 | 1151 | AGCAGCCATGAGGGTGGTGAG |
| Sme2.5_02766.1_g00005.1 | AP2-EREBP | MP00302 | Sme2.5_00096.1_g00007.1 | 3GT | 2.74E-05 | 0.0883 | 489 | 509 | GTGGGCTCCGGAGTAGGTGAT |
| Sme2.5_02766.1_g00005.1 | AP2-EREBP | MP00302 | Sme2.5_00228.1_g00013.1 | 3GT | 0.000043 | 0.0972 | 549 | 569 | GGATCAGTTGCAGGAGGAGAA |
| Sme2.5_02766.1_g00005.1 | AP2-EREBP | MP00302 | Sme2.5_00228.1_g00013.1 | 3GT | 5.21E-05 | 0.101 | 21 | 41 | AGCGAGGTTGCAGGAGGAGAA |
| Sme2.5_02766.1_g00005.1 | AP2-EREBP | MP00302 | Sme2.5_00228.1_g00013.1 | 3GT | 5.21E-05 | 0.101 | 90 | 110 | AGCGAGGTTGCAGGAGGAGAA |
| Sme2.5_02766.1_g00005.1 | AP2-EREBP | MP00302 | Sme2.5_00228.1_g00013.1 | 3GT | 5.31E-05 | 0.101 | 730 | 750 | AAAGAAGACGAGGATGGTGGG |
| Sme2.5_02766.1_g00005.1 | AP2-EREBP | MP00302 | Sme2.5_00228.1_g00013.1 | 3GT | 0.000079 | 0.133 | 441 | 461 | AGCAAAGTTGCAGGAGGAGAA |
| Sme2.5_02766.1_g00005.1 | AP2-EREBP | MP00302 | Sme2.5_00228.1_g00013.1 | 3GT | 8.83E-05 | 0.138 | 879 | 899 | GGGAAGTGTGGAGGTGGCTCC |
| Sme2.5_02766.1_g00005.1 | AP2-EREBP | MP00302 | Sme2.5_00228.1_g00013.1 | 3GT | 9.46E-05 | 0.143 | 510 | 530 | GGATCGGTTGAAGGAGGAGAA |
| Sme2.5_02766.1_g00005.1 | AP2-EREBP | MP00302 | Sme2.5_02148.1_g00009.1 | 5GT | 4.36E-05 | 0.0972 | 1438 | 1458 | TTTTGGGTGGGTGGTGGAGGG |
| Sme2.5_02766.1_g00005.1 | AP2-EREBP | MP00302 | Sme2.5_01638.1_g00005.1 | ANS | 2.8E-06 | 0.038 | 1046 | 1066 | AGAGGTGAGATTGGTGGTGCT |
| Sme2.5_02766.1_g00005.1 | AP2-EREBP | MP00302 | Sme2.5_01193.1_g00009.1 | CHI | 1.82E-05 | 0.0657 | 717 | 737 | AGATCAGGTACTGGAGGAGGG |
| Sme2.5_02766.1_g00005.1 | AP2-EREBP | MP00302 | Sme2.5_01193.1_g00009.1 | CHI | 5.41E-05 | 0.101 | 1313 | 1333 | TTGGGTGGTTTAAATGGCGGC |
| Sme2.5_02766.1_g00005.1 | AP2-EREBP | MP00302 | Sme2.5_01193.1_g00009.1 | CHI | 5.41E-05 | 0.101 | 1325 | 1345 | TTGGGTGGTTTAAATGGCGGC |
| Sme2.5_02766.1_g00005.1 | AP2-EREBP | MP00302 | Sme2.5_02154.1_g00001.1 | CHS | 4.96E-06 | 0.0402 | 1122 | 1142 | GTAGGTAGCATTAGTGGCGGA |
| Sme2.5_02766.1_g00005.1 | AP2-EREBP | MP00302 | Sme2.5_02154.1_g00001.1 | CHS | 9.21E-06 | 0.0483 | 1125 | 1145 | GGTAGCATTAGTGGCGGATGG |
| Sme2.5_02766.1_g00005.1 | AP2-EREBP | MP00302 | Sme2.5_04927.1_g00001.1 | F3'H | 6.26E-05 | 0.109 | 98 | 118 | TCAGCTGGTGGAAATGGATGA |
| Sme2.5_02766.1_g00005.1 | AP2-EREBP | MP00302 | Sme2.5_04927.1_g00001.1 | F3'H | 6.26E-05 | 0.109 | 1237 | 1257 | TCAGCTGGTGGAAATGGATGA |
| Sme2.5_02766.1_g00005.1 | AP2-EREBP | MP00302 | Sme2.5_01638.1_g00003.1 | LDOX | 6.99E-08 | 0.00624 | 508 | 528 | TCCGATGGGGGAGGTGGTGGA |
| Sme2.5_02766.1_g00005.1 | AP2-EREBP | MP00302 | Sme2.5_01638.1_g00003.1 | LDOX | 4.56E-07 | 0.0136 | 511 | 531 | GATGGGGGAGGTGGTGGAGCT |
| Sme2.5_02766.1_g00005.1 | AP2-EREBP | MP00302 | Sme2.5_01638.1_g00003.1 | LDOX | 4.7E-06 | 0.0402 | 573 | 593 | AGGGAGGGGAGAGGCGGTGAT |
| Sme2.5_02766.1_g00005.1 | AP2-EREBP | MP00302 | Sme2.5_01638.1_g00003.1 | LDOX | 9.82E-06 | 0.0487 | 585 | 605 | GGCGGTGATGGAAGCGTCCGG |
| Sme2.5_02766.1_g00005.1 | AP2-EREBP | MP00302 | Sme2.5_01638.1_g00003.1 | LDOX | 1.84E-05 | 0.0657 | 540 | 560 | CGTATTGGAGGTGATGGCTGT |
| Sme2.5_02766.1_g00005.1 | AP2-EREBP | MP00302 | Sme2.5_01638.1_g00003.1 | LDOX | 2.77E-05 | 0.0883 | 534 | 554 | AGATGTCGTATTGGAGGTGAT |
| Sme2.5_02766.1_g00005.1 | AP2-EREBP | MP00302 | Sme2.5_01638.1_g00003.1 | LDOX | 4.14E-05 | 0.0972 | 505 | 525 | TTGTCCGATGGGGGAGGTGGT |
| Sme2.5_02766.1_g00005.1 | AP2-EREBP | MP00302 | Sme2.5_01638.1_g00003.1 | LDOX | 8.28E-05 | 0.134 | 579 | 599 | GGGAGAGGCGGTGATGGAAGC |
| Sme2.5_02766.1_g00005.1 | AP2-EREBP | MP00302 | Sme2.5_01638.1_g00003.1 | LDOX | 8.72E-05 | 0.138 | 570 | 590 | AGAAGGGAGGGGAGAGGCGGT |
| Sme2.5_02766.1_g00005.1 | AP2-EREBP | MP00302 | Sme2.5_16832.1_g00002.1 | PAL | 3.96E-07 | 0.0136 | 334 | 354 | GGGGGGTGGGGGGGCGGAGGG |
| Sme2.5_02766.1_g00005.1 | AP2-EREBP | MP00302 | Sme2.5_16832.1_g00002.1 | PAL | 3.65E-05 | 0.0972 | 353 | 373 | GGTGTGGACTTTAGTGGAGGC |
| Sme2.5_02766.1_g00005.1 | AP2-EREBP | MP00302 | Sme2.5_16832.1_g00002.1 | PAL | 3.68E-05 | 0.0972 | 1387 | 1407 | GTAGATGGGATTTGTGGTGAG |
| Sme2.5_02766.1_g00005.1 | AP2-EREBP | MP00302 | Sme2.5_16832.1_g00002.1 | PAL | 5.15E-05 | 0.101 | 1178 | 1198 | AAAAGTTTCGGGGTTGGTGGA |
| Sme2.5_02766.1_g00005.1 | AP2-EREBP | MP00302 | Sme2.5_00864.1_g00009.1 | PAL | 4.64E-05 | 0.101 | 1284 | 1304 | GCAGGGGAATGATGAGGCGGG |
| Sme2.5_02766.1_g00005.1 | AP2-EREBP | MP00302 | Sme2.5_11682.1_g00004.1 | PAL | 0.000017 | 0.0657 | 48 | 68 | GGATACGGCGGGGTCGGTTCG |
