# Supplementary material for: Comparative transcription analysis of photosensitive and non-photosensitive eggplants to identify genes involved in dark regulated anthocyanin synthesis
Source: BMC Genomics. 2019 Aug 28;20:678. doi: 10.1186/s12864-019-6023-4 (PMC6712802; doi:10.1186/s12864-019-6023-4)
Supplement: Supplementary file 7 — Table S6. The interaction score of transcription factors in Fig. 9. (DOCX 13 kb) [file 12864_2019_6023_MOESM7_ESM.docx]

| Source name | Target name | Score |
| --- | --- | --- |
| MYB4 | TT8 | 0.994 |
| TCP | C2C2-Dof | 0.952 |
| C2C2-YABBY | BIM1 | 0.94 |
| TT8 | MYB113 | 0.914 |
| TT8 | MYC2 | 0.908 |
| TTG2 | C2C2-YABBY | 0.872 |
| TCP | C2C2-YABBY | 0.742 |
| TCP | C2C2-CO-like | 0.722 |
| ICE1 | C2C2-YABBY | 0.686 |
| MYB15 | MYC2 | 0.646 |
| MYB113 | MYC2 | 0.606 |
| BIM1 | MYC2 | 0.604 |
| ICE1 | MYB15 | 0.5 |

**Table S6 The interaction score of transcription factors in figure 9**
