# Supplementary material for: Comparative transcription analysis of photosensitive and non-photosensitive eggplants to identify genes involved in dark regulated anthocyanin synthesis
Source: BMC Genomics. 2019 Aug 28;20:678. doi: 10.1186/s12864-019-6023-4 (PMC6712802; doi:10.1186/s12864-019-6023-4)
Supplement: Supplementary file 8 — Table S1. List of all the primer sequences used in this study. (DOCX 15 kb) [file 12864_2019_6023_MOESM8_ESM.docx]

**Table S1 List of all the primer sequences used in this study**

| Gene | Forward primer (5’-3’) | Reverse primer (5’-3’) |
| --- | --- | --- |
| For real-time qPCR |  |  |
| MYB1(Sme2.5_05099.1_g00002.1) | AGTGGCATCTTGTTCCTGCT | ATCCACTTCATCCGAAGCAA |
| HY5(Sme2.5_03211.1_g00004.1) | CAAGTTCCATTGCGGCTAGT | CTCCGGCACTCTTCTGATCT |
| WD40(Sme2.5_04405.1_g00003.1) | ATGAGATTGCATGGAACACG | CAACCAGCTGTGTGAGCATT |
| CHI(Sme2.5_01193.1_g00009.1) | ATGGTGAAGCCTGTTGGTTC | GGAATTGCACTCTCCTCCAA |
| DFR(Sme2.5_01401.1_g00004.1) | CATTGAGACTTGCCGACAGA | ATTCTCCTTGCCACTTGCAT |
| F3H(Sme2.5_00015.1_g00020.1) | GCCTTAACCAAGGCATGTGT | TGATGGTTCCTGGATCAGTG |
| WD40(Sme2.5_05196.1_g00001.1) | AATCGGTCTGGAATGCTCAC | ATCTTCATGTCTGCCGAACC |
| TT8(Sme2.5_00592.1_g00005.1) | CGAGGACGAGGAAGAAGATG | GCCAAGCCGTATAGCTTCAG |
| MYB73(Sme2.5_24183.1_g00002.1) | CTTCTTAATGGCCGGACTGA | ATCGGCGAGTTCGTTACCTT |
| bHLH(Sme2.5_01795.1_g00002.1) | GCTTCCACTGTAACGGCTTC | TAGCGTAGGTCCACGTCTCC |
| *Smactin* | GTCGGAATGGGACAGAAGGATG | GTGCCTCAGTCAGGAGAACAGGGT |
| For yeast one-hybrid |  |  |
| YABBY-pB42AD (Sme2.5_03738.1_g00001.1) | TGCCTCTCCCGAATTCATGTCATCTTCATCTGCTCCGGACC | TCCAAAGCTTCTCGAGTTAGTAAGGAGCTACACCAATGTTT |
| MYB94-pB42AD (Sme2.5_00669.1_g00015.1) | TGCCTCTCCCGAATTCATGGGAAGACCACCTTGTTGTGATAAA | TCCAAAGCTTCTCGAGTCAAAAGAAGTCAGCAGATTCACCC |
| HD-pB42AD (Sme2.5_02806.1_g00001.1) | TGCCTCTCCCGAATTCATGGACTTAACCAACAACACAGCTA | TCCAAAGCTTCTCGAGTCAAGATGAAGAAGAAGACCCATTG |
| BIM1-pB42AD (Sme2.5_00537.1_g00003.1) | TGCCTCTCCCGAATTCATGGAGCTGCCTCAGCCCAGA | TCCAAAGCTTCTCGAGTTAGTTTGTTTTCCGTCTTTTGAAG |
| MYB19-pB42AD (Sme2.5_00962.1_g00008.1) | TGCCTCTCCCGAATTCATGGTTAGAGCTCCTTGTTGTGA | TCCAAAGCTTCTCGAGTCAAAATTCTGGTAACTCAAATAAG |
| AP2-pB42AD (Sme2.5_02766.1_g00005.1) | TGCCTCTCCCGAATTCATGGCTATTGTGGATCAAGGTGCTA | TCCAAAGCTTCTCGAGTCAAGCCCCAAGTTCAGAATCCA |
| MYC2-pB42AD (Sme2.5_02104.1_g00004.1) | TGCCTCTCCCGAATTCATGGAACTGACGGAGTATAGATTGT | TCCAAAGCTTCTCGAGTCGCGATTCAGCAATTTTTGAA |
| TTG2-pB42AD (Sme2.5_02680.1_g00006.1) | TGCCTCTCCCGAATTCATGGAGGTCAATGAAGCAGCA | TCCAAAGCTTCTCGAGCTATGATTTCTCTTTCATAGATGAA |
| ICE1-pB42AD (Sme2.5_06398.1_g00001.1) | TGCCTCTCCCGAATTCATGTTGTCTGGGGTGAATGGTTCTT | TCCAAAGCTTCTCGAGTTAAATCATCCCCCCATGGAAGCCA |
| TT8-pB42AD (Sme2.5_00592.1_g00005.1) | TGCCTCTCCCGAATTCATGGAGATTATACAGCCTAATAGC | TCCAAAGCTTCTCGAGTTAATTAACTCTAGGGATTATCTGA |
| 3GT-PlacZi (Sme2.5_00228.1_g00013.1) | TATTGGATCGGAATTCATGGTTTGGTGGATTTAATCTATAT | GAGCACATGCCTCGAGCTGTAACTTCAGGTGGAATGTCA |
| TT8-PlacZi (Sme2.5_00592.1_g00005.1) | TATTGGATCGGAATTCATTTTTATACCAACTATATTTATTT | GAGCACATGCCTCGAGTATTATTTTTAATTTGACTTGAAAT |
| CHS-PlacZi (Sme2.5_13923.1_g00001.1) | CCGGAATTCTGAAGGTTAAATATGTTGAGTCATAT | CCGCTCGAGTTTCGCCCGAAAAAATGGTGAATA |
| ANS-PlacZi (Sme2.5_01638.1_g00005.1) | CCGGAATTCCGTCGAGAGAGATGAAGAAGAAAA | CCGCTCGAGCTCTTTAACGCGGAGTACTTATTTAG |
